# Diet-induced obesity alters the ovarian chemical biotransformation and oxidative stress response proteins both basally and in response to 7,12-dimethylbenz[a]anthracene exposure

**DOI:** 10.1093/toxsci/kfae150

**Published:** 2025-02-06

**Authors:** Kelsey Timme, Imaobong Inyang, Hunter E White, Aileen F Keating

**Affiliations:** Department of Animal Science, Iowa State University, Ames, IA 50011, United States; Department of Animal Science, Iowa State University, Ames, IA 50011, United States; Department of Animal Science, Iowa State University, Ames, IA 50011, United States; Department of Animal Science, Iowa State University, Ames, IA 50011, United States

**Keywords:** obesity, ovary, 7,12-dimethylbenz[a]anthracene, ovarian proteome

## Abstract

7,12-Dimethylbenz[a]anthracene (DMBA) is a polycyclic aromatic hydrocarbon that causes female infertility via DNA damage, and the ovary has the capacity to mitigate DMBA exposure via the action of proteins including the glutathione *S*-transferase (GST) family. Due to previous findings of DNA damage and a reduced ovarian chemical biotransformation response to DMBA exposure in hyperphagia-induced obese mice, this study investigated the hypothesis that diet-induced obesity would hamper the ovarian biotransformative response to DMBA exposure. Six-week-old C57BL6/J mice were fed either a normal rodent diet (L) or a high fat high sucrose diet (O) until the O group was ∼30% heavier than the L. Both L and O mice were exposed to either corn oil (C) or DMBA (1 mg/kg) for 7 d. Liver weight was increased (*P *<* *0.05) in obese mice exposed to DMBA but no effect on spleen weight, uterine weight, ovary weight, estrous cyclicity, or circulating 17β-estradiol and progesterone were observed. Primordial and preantral follicle numbers were higher (*P *<* *0.05) in the obese mice and there was a tendency (*P *=* *0.055) for higher antral follicles in DMBA-exposed obese mice. The ovarian proteome was identified by LC-MS/MS analysis to be altered both by diet-induced obesity and by DMBA exposure with changes observed in levels of proteins involved in oocyte development and chemical biotransformation, including GST isoform pi. Fewer proteins were affected by the combined exposure of diet and DMBA than by a single treatment, indicating that physiological status impacts the response to DMBA exposure.

Approximately 41% of reproductive-age women in the United States are overweight or obese (Hales et al. 2020). Although obesity is caused by a variety of factors, a major influence is dietary fat intake (Lin and Li 2021). The recommended consumption of dietary fat is ∼20% to 35% of daily calories for adult women (Vannice and Rasmussen 2014), whereas a high fat diet varies from 30% to 50% of caloric intake as fat (Schwingshackl and Hoffmann 2013). Individuals classified as obese have a body mass index ≥30 and have an increased likelihood of developing health conditions including type II diabetes, cardiovascular disease, and nonalcoholic fatty liver disease compared with lean counterparts (Sun et al. 2022). Reproductive-age obese women also experience lower fertility rates which span processes including hormonal imbalance (Lin et al. 2009), irregular menstrual cyclicity (Wei et al. 2009), higher rates of anovulation (Fichman et al. 2020), increased time to conception (Gesink Law et al. 2007), and higher risk of pregnancy complications (Raatikainen et al. 2006).

7,12-Dimethylbenz[a]anthracene (DMBA) is a model ovarian and genetic toxicant that is a polycyclic aromatic hydrocarbon (PAH). The PAH chemicals are produced through the combustion of organic material like wildfires, and from industrial production such as biofuels and cigarette tar (Estrellan and Iino 2010). DMBA undergoes bioactivation in the liver and ovary through a 3-step process involving the enzymes cytochrome P450 (CYP) isoform 1B1 (CYP1B1), microsomal epoxide hydrolase (EPHX1), and CYP isoform 1A1 (CYP1A1) producing the ovotoxic metabolite DMBA-3,4-diol-1,2 epoxide (Rajapaksa et al. 2007). PAHs, including benz[a]anthracene, benzo[a]pyrene, chrysene have been identified in follicular fluid, placental tissue, and breast milk (Martin Santos et al. 2019) and result in female infertility through DNA damage (Ganesan et al. 2014).

Although obesity and DMBA induce individual ovarian effects, obesity alters the ovarian response to DMBA exposure in mice, suggesting that these 2 stressors may have an interactive effect on ovotoxicity. Obesity has been shown to attenuate the response of ovarian DNA damage repair proteins (Ganesan et al. 2014), ovarian gap junction proteins (Ganesan et al. 2015), and chemical bioactivation enzymes (Nteeba et al. 2014a) to DMBA exposure.

The glutathione *S*-transferase (GST) family are biotransformation enzymes capable of detoxifying endogenous and environmental chemicals including carcinogens (Strange et al. 2001), or metabolites of these compounds, through the conjugation of an electrophilic xenobiotic compound with reduced glutathione (GSH) in the Phase II biotransformation reaction (Rahilly et al. 1991). Our previous research into the combined effects of genetic-induced obesity utilizing KK.CG-A^y/^J lethal yellow mice and environmental toxicant exposures including DMBA have discovered an alteration in the abundance of GST enzymes, particularly the π (GSTP) isoform (Nteeba et al. 2014a). GSTP is documented to be involved in both the ovarian response to DMBA exposure (Bhattacharya and Keating 2012) and detoxification of DMBA (Henderson et al. 1998), thus an obesity-induced change in ovarian GSTP raises concern that ovotoxicity might be impacted in the obese female.

Based upon our previous findings of DNA damage and a reduced ovarian protective response to ovotoxicant exposure in hyperphagia-induced obese relative to lean mice (Nteeba et al. 2014a), this study investigated the hypothesis that diet-induced obesity would also hamper the ovarian biotransformative response to DMBA exposure.

## Materials and methods

### Reagents

DMBA (purity ≥95%) (CAS No. D3254), Paraformaldehyde (P6148), Mayer’s hematoxylin solution (MHS32), Eosin Y disodium salt (E4382), Trizma hydrochloride (T3253), and ethylenediaminetetraacetic acid (EDTA) (EDS-100G) were purchased from Sigma-Adrich Inc (St Louis, MO, United States). 10× Phosphate-buffered saline (BP665) was obtained from Fisher BioReagents (Fair Lawn, NJ, United States). Citrisolv (1601) was procured from Decon Labs Inc (King of Prussia, PA, United States). Tert-butanol (A401-1), paraffin (P31-500), Superfrost Plus microscope slides (12-550-15), and permount mounting medium (SP15-500) were purchased from Thermo Fisher Scientific (Waltham, MA, United States). Platinum line coverglass (71887-23) was procured from Electron Microscopy Sciences (Hatfield, PA, United States). A phosphatase and protease inhibitor cocktail (5872S) was obtained from Cell Signaling Technology (Danvers, MA, United States). Bicinchoninic Acid Assay (BCA) protein assay kit (23225) was purchased from Thermo Scientific (Rockfield, IL, United States).

### Animal experimental design

The experimental animal protocols for this study were approved by the Iowa State University Institutional Animal Care and Use Committee. Female wild-type C57BL6J (000664; *n* = 52) were purchased from Jackson Laboratories (Bar Harbor, Maine) at 5 wk of age. The mice were housed in Innovive cages with 5 animals per cage. The room was maintained under control conditions with a temperature of 22 °C and a 12-h light/dark cycle. The mice had ad libitum access to water and food (2014 Teklad Global 14% Protein Rodent Diet [3.7% fat, 0% sucrose]). Beginning at 6 wk of age, the mice were divided into 2 groups (*n* = 30 per diet) and continuously fed either the 14% Protein Rodent Diet as the lean diet group, whereas the second set of mice was fed a diet consisting of 45% fat and 20% sucrose (D12451, Research Diets) as the high fat high sucrose (HFHS) diet group. Mouse weight and diet consumption were monitored weekly. HFHS diet was replaced following each weight check to ensure freshness and avoid rancidity. At 12 wk of age, the HFHS was 30% heavier in body mass than their lean diet counterparts which was an important consideration because in the hyperphagia-induced obesity model, both impaired fasting glucose and primordial follicle loss were observed at a similar body weight increase (Nteeba et al. 2014b). HFHS mice who did not achieve a 30% increase in body weight were excluded from the study.

Exposure to 1 mg/kg DMBA for 14 d caused loss of almost the entirety of ovarian follicles (Nteeba et al. 2014b). Since a goal of this study was to investigate molecular alterations preceding ovarian cell death, the exposure duration was shortened to 7 d. This exposure length ensured that an entire estrous cycle was completed. Each diet group was divided, and mice were dosed with either corn oil (CO) as a vehicle control or 1 mg/kg of body weight of DMBA intraperitoneally once a day for 7 d to ensure that a complete estrous cycle was completed during exposure. Thus, the 4 experimental groups were Lean Corn Oil (LC; *n* = 11), Lean DMBA (LD; *n* = 11), Obese Corn Oil (OC; *n* = 12), and Obese DMBA (OD; *n* = 10).

### Monitoring of estrous cyclicity

Vaginal cytology was performed beginning with the first day of intraperitoneal injection and continued until euthanasia. The vagina was gently lavaged with 50 µl 1× PBS using sterile plastic pipette tips. The resulting flushed cells were observed under a microscope at 40× magnification. Proestrus is characterized by the majority of nucleated epithelial cells in the sample. In the estrus stage, the cornified cells are large, irregularly shaped, and contain no visible nucleus. Leukocytes are present in metestrus and diestrus with an equal mix of leukocytes to epithelial cells in metestrus compared with a majority of leukocytes in diestrus (Cora et al. 2015).

### Tissue collection

Euthanasia occurred on the second day of diestrus of the estrous cycle following completion of the 7 d of exposure and was performed by CO_2_ asphyxiation followed by cervical dislocation. Cardiac puncture was utilized to collect blood samples. A final body weight was recorded. Ovaries, uterus, spleen, and liver were collected and cleaned of excess fat and weighed. One ovary was flash frozen in liquid nitrogen and stored at −80 °C for protein analysis, whereas the second was fixed in 4% paraformaldehyde and embedded in paraffin wax for sectioning and follicle quantification.

### Serum 17β-estradiol and progesterone hormone level quantification

A blood sample was collected utilizing cardiac puncture immediately following euthanasia. The blood was allowed to clot at room temperature for 15 min, then transferred to ice. The clot was then removed by centrifuge at 1,500×*g* at 4 °C for 10 min. Serum was transferred to a new tube and stored at −20 °C. Serum was shipped to the Ligand Assay and Analysis Core Library at the University of Virginia for analysis of 17β-estradiol and progesterone through ELISA. For the 17β-estradiol assay, multiple samples were determined to be below the assay limit of detection (LOD) (LC *n* = 5 of 8; LD *n* = 4 of 8; OC *n* = 4 of 8; and OD *n* = 5 of 8) and for those samples a value of LOD/SQRT(2) was inputted for statistical analysis. For the progesterone assay, 2 samples in the LC group had a high CV value, thus were not included in the statistical analysis.

### Ovarian follicle composition enumeration

One ovary from each mouse was preserved in 4% paraformaldehyde for 24 h, then transferred to 70% ethanol. The ovaries were then embedded in paraffin wax and sectioned into 5 µm slices (*n* = 6 per treatment). Every sixth section was mounted onto a microscope slide. The paraffin wax was removed and ovarian sections were stained with hematoxylin and 1% eosin. Follicles were counted using an Olympus CX43 microscope in every second mounted section, equating to every 60 µm in the ovary to prevent double counting. Follicle classification was based on the procedure described by Flaws et al. (1994). Unhealthy follicles were identified if they have granulosa cell pyknosis and intense oocyte eosinophilic staining (Devine et al. 2002). Follicles with misshapen oocytes or deteriorated granulosa and theca cells were also considered atretic. A follicle was considered healthy if it contained a distinct oocyte nucleus. A follicle was classified as a primordial follicle if its nucleated oocyte was surrounded by a partial or complete layer of squamous granulosa cells. A primary follicle contained a single layer of cuboidal granulosa cells surrounding its oocyte. A follicle whose nucleated oocyte was surrounded by multiple layers of granulosa cells was classified as a secondary follicle. A follicle was classified as a preovulatory follicle if its nucleated oocyte was surrounded by at least 2 layers of granulosa cells and a fluid-filled antrum. Corpora lutea were distinguished by a membrane-enclosed, oocyte-devoid area. Values represent a total number of each follicle type per ovary.

### Ovarian protein isolation and LC-MS/MS analysis

Total ovarian protein was isolated from one flash-frozen ovary per mouse (*n* = 5 per treatment) using Tris–HCL and EDTA. Protein was quantified using the Pierce BCA Protein Assay Kit. Whole ovary protein extract was sent to the Protein Facility of the Iowa State University Office of Biotechnology for Q Exactive Tandem Mass Spectrometry LC-MS/MS analysis.

Briefly, the protein was reduced by dithiothreitol, followed by the modification of cysteine groups by iodoacetamide. Trypsin/Lys-C digestion occurred overnight and was halted the next day by formic acid. Samples were then dried in a SpeedVac chamber. The proteins were desalted with a C18 MicroSpin Column (Nest Group SEM SS18V) followed by repeated drying within the SpeedVac Chamber. The EASY nLC-1200 Column was utilized to separate the peptides as described previously (Novbatova et al. 2023). The fragments were evaluated by Q Exactive Hybrid Quadrupole-Orbitrap Mass Spectrometer with an HCD fragmentation cell. Intact fragments were compared with MASCOT or Sequest HT for protein identification (Xia and Wishart 2016). Normalization was performed using the Peptide Retention Time Calibration standard (Pierce Part No. 88320) as the internal control.

The Genome Informatics Facility at Iowa State University analyzed the protein abundance comparisons. DAVID v 6.8 software was utilized to identify biological pathways altered between treatment groups.

### Proteomic analysis through Stringdb and Cytoscape

Ovarian protein alterations were analyzed through 4 treatment comparisons: LC vs LD, LC vs OC, LD vs OD, and OC vs OD. Proteins that had a *P *<* *0.05 (adjusted for multiple comparisons) were analyzed with Stringdb multiprotein analysis to observe known and potential protein interactions (Szklarczyk et al. 2023). The same proteins were analyzed through Cytoscape (Bindea et al. 2013) and were grouped into biological process pathways through the ClueGo application within Cytoscape (Shannon et al. 2003). Pathways with only one protein identified as being altered, or pathways explicit to organs other than the ovary were removed from this analysis. Each protein pathway was identified by name, the number of proteins altered, and the total number of proteins in that pathway. Proteins with *P *<* *0.1 (adjusted for multiple comparisons) were used for pathway analysis to increase the protein number from which to build pathways of interest. Analysis with the Iregulon tool in Cytoscape was performed to determine transcription factors (TFs) regulating DEPs in treated groups. TFs with a normalized enrichment score (NES) above 5.0 were identified, with a minimum NES above 5.0 being considered highly significant (Verfaillie et al. 2015).

### Statistical analysis

Statistical analysis was conducted with 2-way ANOVA test with Tukey’s correction for multiple comparisons through Prism 9.0.1 software (Graphpad Prism). The results are displayed in bar charts by mean±SEM. For protein network analysis with Stringdb, a medium confidence score of 0.400 and FDR<0.05 was considered significant, whereas multiple testing corrections for enrichment were determined using the Benjamini–Hochberg test procedure for false discovery rate in Cytoscape. A significant difference between treatments was defined as *P *≤* *0.05 and a trend toward a difference was considered if *P *≤* *0.1.

## Results

### Impact of diet-induced and DMBA exposure on food intake and body weight

**Fig. 1.**
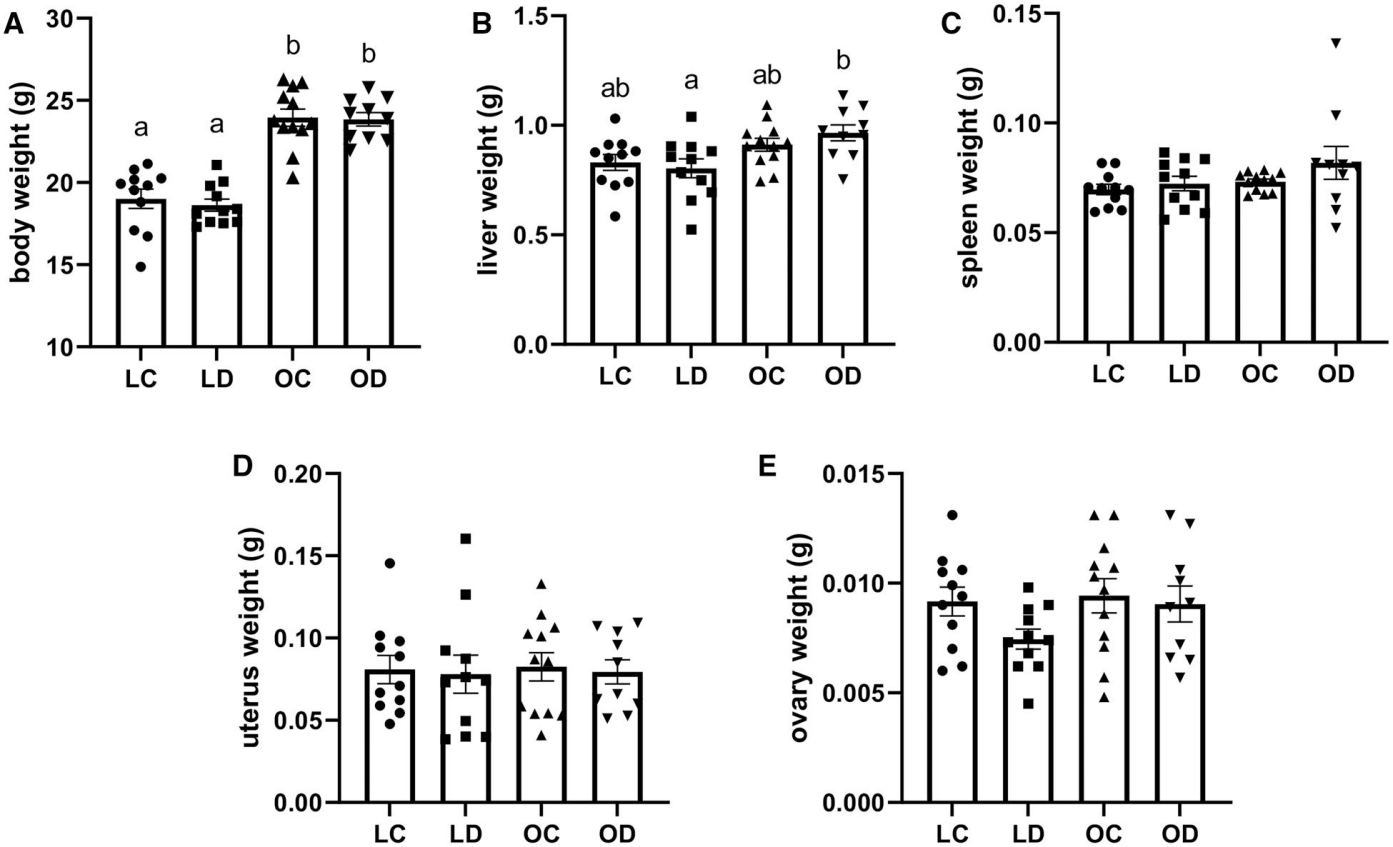
Impact of DMBA exposure on organ weight in chow and HFHS-fed mice. Mice were fed either a chow diet and designated lean (L) or an HFHS diet and indicated as obese (O). Mice were exposed by intraperitoneal injection with corn oil (C) or DMBA (1 mg/kg) for 7 d. Weights of (A) body, (B) liver, (C) spleen, (D) uterus, and (E) ovary were recorded. LC *n* = 11; LD *n* = 11; OC *n* = 12; OD *n* = 10. Bars represent mean values±SEM. Different letters dictate statistical differences at *P *<* *0.05.

Food intake was monitored weekly and measured by consumption per cage. There was no difference in food consumption between lean diet and HFHS diet-fed mice throughout the experiment (data not shown). Mouse body weight was monitored weekly and at euthanasia, the HFHS mice were heavier (*P *<* *0.05; interaction effect *P *=* *0.78; DMBA effect *P *=* *0.62; diet effect *P *<* *0.0001) than the lean mice, with no body weight differences due to DMBA exposure (Fig. 1A).

### Effect of diet-induced obesity and DMBA exposure on organ weight

Immediately following euthanasia, weights of the liver (Fig. 1B), spleen (Fig. 1C), uterus (Fig. 1D), and ovaries (Fig. 1E) were collected. In mice exposed to DMBA, the liver was increased (*P *<* *0.05; interaction: *P *=* *0.28; DMBA effect; *P *=* *0.71; diet effect; *P *=* *0.0018) in weight due to HFHS diet compared with lean diet-fed mice. There was no difference in liver weight in control-treated mice regardless of diet. There was also no difference due to diet, DMBA treatment, or combination of diet and DMBA on the spleen (Interaction; *P *=* *0.46; DMBA effect; *P *=* *0.17; diet effect; *P *=* *0.11), uterus (Interaction; *P *=* *0.99; DMBA effect; *P *=* *0.75; diet effect; *P *=* *0.87), or ovary (Interaction; *P *=* *0.34; DMBA effect; *P *=* *0.14; diet effect; *P *=* *0.19) weights.

### Influence of diet-induced obesity and DMBA exposure on estrous cyclicity and circulating steroid hormone levels

**Fig. 2.**
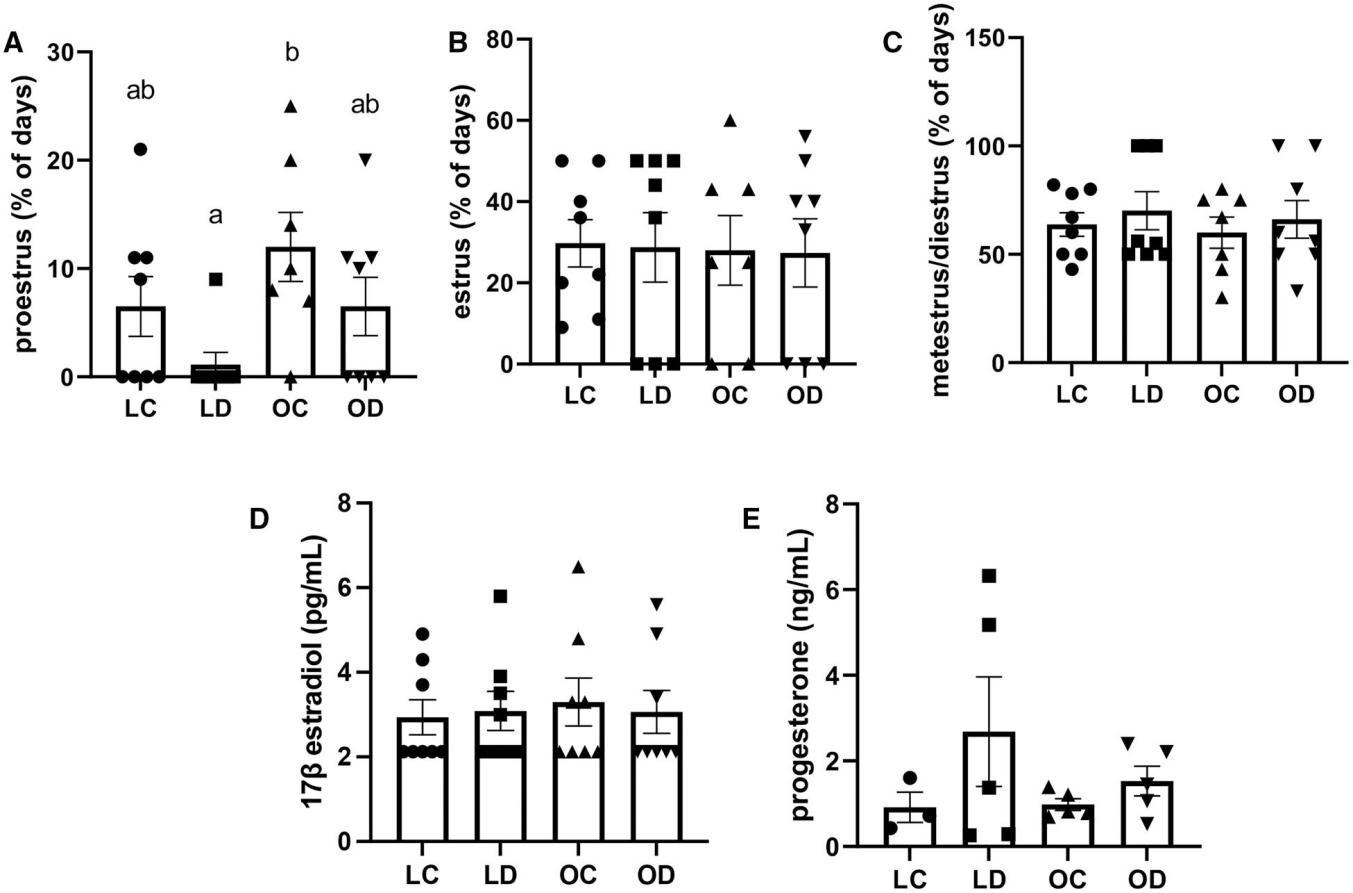
Effect of DMBA exposure on estrous cyclicity and ovarian steroid hormones in circulation in lean and HFHS-fed mice. Mice were fed either a chow diet and were designated lean (L) or an HFHS diet and indicated as obese (O). Mice were exposed by intraperitoneal injection with corn oil (C) or DMBA (1 mg/kg) for 7 d. Estrous cyclicity was monitored by vaginal cytology to determine the percentage of time spent in (A) proestrus, (B) estrus, (C) metestrus/diestrus (LC *n* = 11; LD *n* = 11; OC *n* = 12; OD *n* = 10). Blood was collected immediately following euthanasia and serum was isolated and (D) 17β-estradiol (LC *n* = 3; LD *n* = 4; OC *n* = 4; OD *n* = 3) and (E) progesterone (LC *n* = 3; LD *n* = 5; OC *n* = 5; OD *n* = 5) were measured by ELISA. Bars represent mean values ± SEM. Different letters dictate statistical differences at *P *<* *0.05.

Vaginal cytology was performed on mice from the first day of injection until euthanasia to monitor the effects of DMBA exposure, diet, or the combined effect of DMBA and diet on the length of time spent in each stage of the estrous cycle. The percentage of time in each stage was calculated with the number of days spent in a stage in the numerator and the total number of days evaluated in the denominator. As expected, mice spent most of the time, ∼60%, at the metestrus and diestrus stages combined, ∼30% of time at the estrus stage and 5% to 15% of time in proestrus. Although variability between groups in the time spent in proestrus was observed, there was no impact (*P *<* *0.05) on diet or DMBA exposure on estrous cyclicity (Proestrus Fig. 2A; interaction; *P *=* *0.33; DMBA effect; *P *=* *0.15; diet effect; *P *=* *0.33. Estrus Fig. 2B; interaction; *P *=* *0.58; DMBA effect; *P *=* *0.89; diet effect; *P *=* *0.78. Metestrus and Diestrus Fig. 2C; interaction; *P *=* *0.27; DMBA effect; *P *=* *0.35; diet effect; *P *=* *0.15).

Many samples had circulating hormone levels that were below the detectable range of the assay and thus were too low to analyze. Levels of 17β-estradiol were measurable in *n* = 3 LC, *n* = 4 LD, *n* = 4 OC, and *n* = 3 OD mice, whereas progesterone was measurable in *n* = 3 LC, *n* = 5 LD, *n* = 5 OC, and *n* = 5 OD mice. There was no impact (*P *<* *0.05) of diet-induced obesity or DMBA exposure on circulating 17β-estradiol or progesterone levels (17β-estradiol Fig. 2D; interaction; *P *=* *0.76; DMBA effect; *P *=* *0.95; diet effect; *P *=* *0.57. Progesterone Fig. 2E; interaction; *P *=* *0.45; DMBA effect; *P *=* *0.16; diet effect; *P *=* *0.49).

### Impact of diet-induced obesity and DMBA exposure on ovarian follicle number

**Fig. 3.**
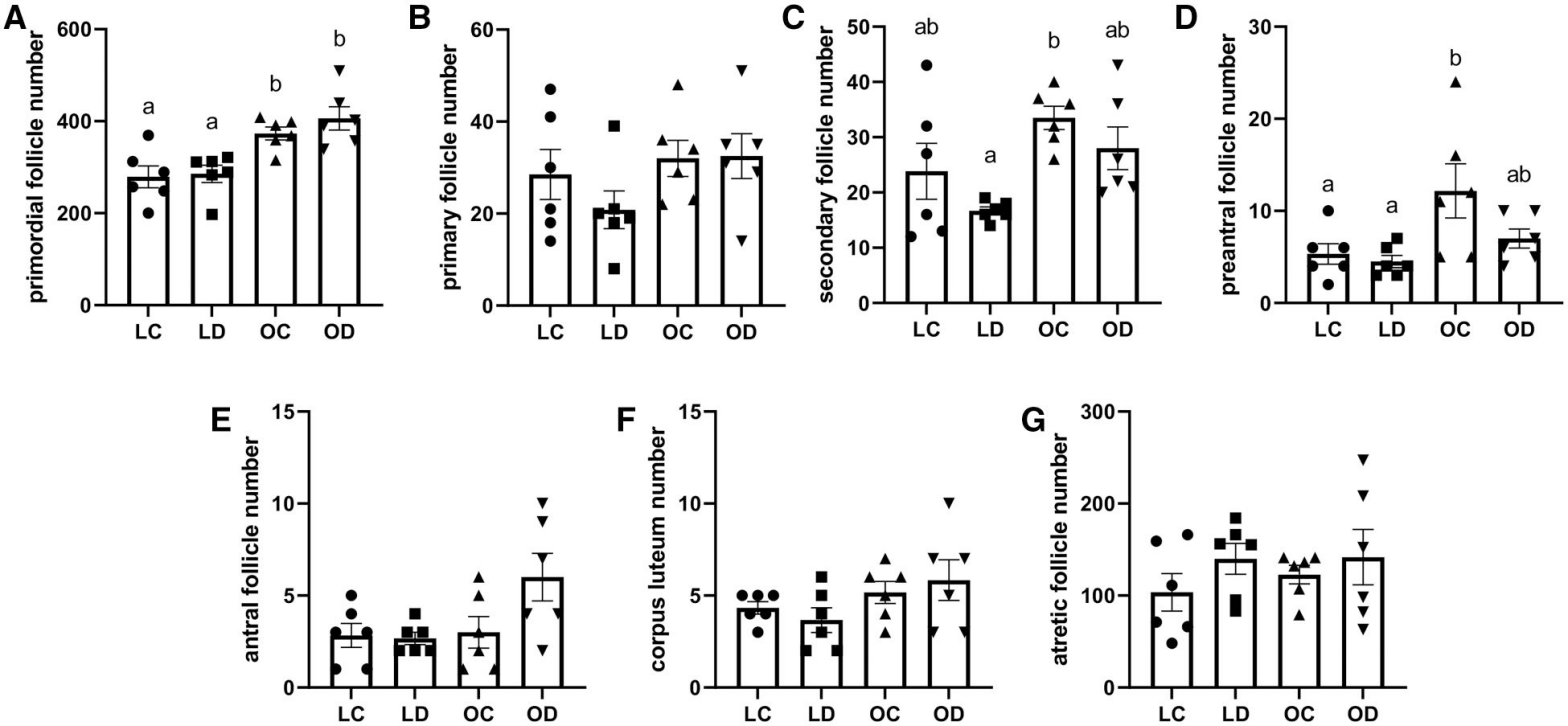
Impact of DMBA exposure on ovarian follicle number in lean and HFHS-fed mice. Ovarian sections were stained with hematoxylin and eosin and the number of (A) primordial follicles, (B) primary follicles, (C) secondary follicles, (D) preantral follicles, (E) antral follicles, (F) corpus luteum, and (G) regressing follicles. *n* = 6 per treatment group. Values represent raw numbers from every 12th section sliced at 5 µm. Bars represent mean values ± SEM. Letters dictate *P *<* *0.05.

Mice who became obese on the HFHS diet had higher numbers of primordial (*P *<* *0.05; Fig. 3A; interaction; *P *=* *0.53; DMBA effect; *P *=* *0.36; diet effect; *P *<* *0.0001), secondary (Interaction; *P *=* *0.81; DMBA effect; *P *=* *0.07; diet effect; *P *=* *0.005), and preantral (*P *<* *0.05; Fig. 3D; interaction; *P *=* *0.21; DMBA effect; *P *=* *0.09; diet effect; *P *=* *0.01) follicles regardless of DMBA exposure. In DMBA-treated mice, those on the HFHS diet had a tendency toward higher antral follicle counts compared with lean mice (*P *=* *0.06; Fig. 3E; interaction; *P *=* *0.08; DMBA effect; *P *=* *0.11; diet effect; *P *=* *0.05), but this difference was not observed between mice in the absence of DMBA exposure. No differences were observed in primary (Interaction; *P *=* *0.39; DMBA effect; *P *=* *0.45; diet effect; *P *=* *0.12), corpora lutea (Interaction; *P *=* *0.37; DMBA effect; *P *=* *0.99; diet effect; *P *=* *0.05), or atretic follicle (Interaction; *P *=* *0.68; DMBA effect; *P *=* *0.20; diet effect; *P *=* *0.62) numbers due to HFHS diet or DMBA exposure (Fig. 3).

### Effect of diet-induced obesity and DMBA treatment on the ovarian proteome

Obesity decreased (*P *<* *0.05) 89 and increased (*P *<* *0.05) 67 proteins compared with controls (Table S1; LC vs OC). In lean mice, DMBA exposure reduced (*P *<* *0.05) 27 and increased (*P *<* *0.05) 73 ovarian proteins compared with controls (Table S2; LC vs LD). In obese mice, DMBA exposure reduced (*P *<* *0.05) 3 and increased (*P *<* *0.05) 9 proteins compared with HFHS-fed mice without DMBA (Table S3; OC vs OD). DMBA exposure decreased (*P *<* *0.05) 16 and increased (*P *<* *0.05) 13 ovarian proteins in the obese exposed relative to the lean identically exposed mice (Table S4; LD vs OD).

### Impact of diet-induced obesity and DMBA treatment on protein pathways

**Fig. 4.**
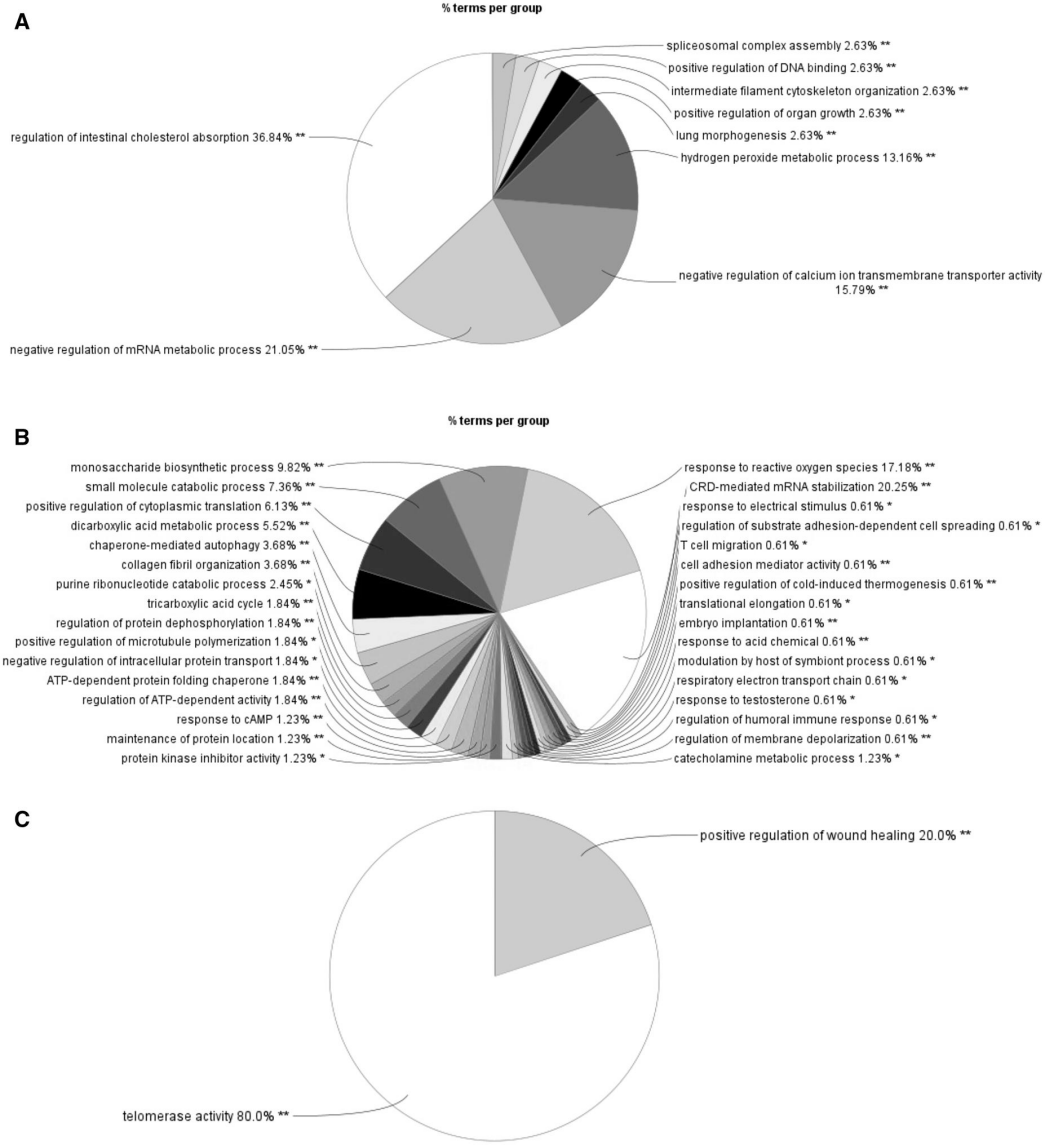
Biological processes altered by DMBA and HFHS diet. Ovarian proteins that differed between treatment groups (*P *<* *0.05) were inputted to Cytoscape. The pie charts represent the number of processes potentially altered within each biological category. (A) LC vs LD, (B) LC vs OC, (C) LD vs OD. OC vs OD contained too few proteins to determine effects on biological processes.

Alterations to protein pathways were analyzed using proteins that were identified to be altered at a statistical significance level of *P *<* *0.1 and adjusted for multiple comparisons to account for the interaction of multiple proteins in a pathway. A pie chart of altered biological processes was produced through Cytoscape (Fig. 4). Each segment of the pie is divided into subcategories of protein pathways with lists of altered proteins within each pathway (Table S5A–C). The size of the pie slice represents the number of subcategories, rather than the number of altered proteins within the biological process. In lean mice, DMBA exposure resulted in 9 altered biological processes, with the largest pie slices representing regulation of cholesterol absorption, negative regulation of mRNA metabolic process, negative regulation of calcium ion transmembrane transporter activity, and hydrogen peroxide metabolic process (Fig. 4A; LC vs LD). Obesity basally altered 32 biological processes, many of which are related to metabolic processes. The largest pie slices represent mRNA stabilization and response to reactive oxygen species (Fig. 4B; LC vs OC). DMBA exposure between obese and lean mice, altered 2 biological processes including telomerase activity and positive regulation of wound healing (Fig. 4C; LD vs OD). In obese mice, there were too few altered proteins at *P *<* *0.05 due to DMBA exposure to identify biological processes altered (OC vs OD).

### String interaction plots of proteins altered by DMBA in lean and HFHS-fed mice

**Fig. 5.**
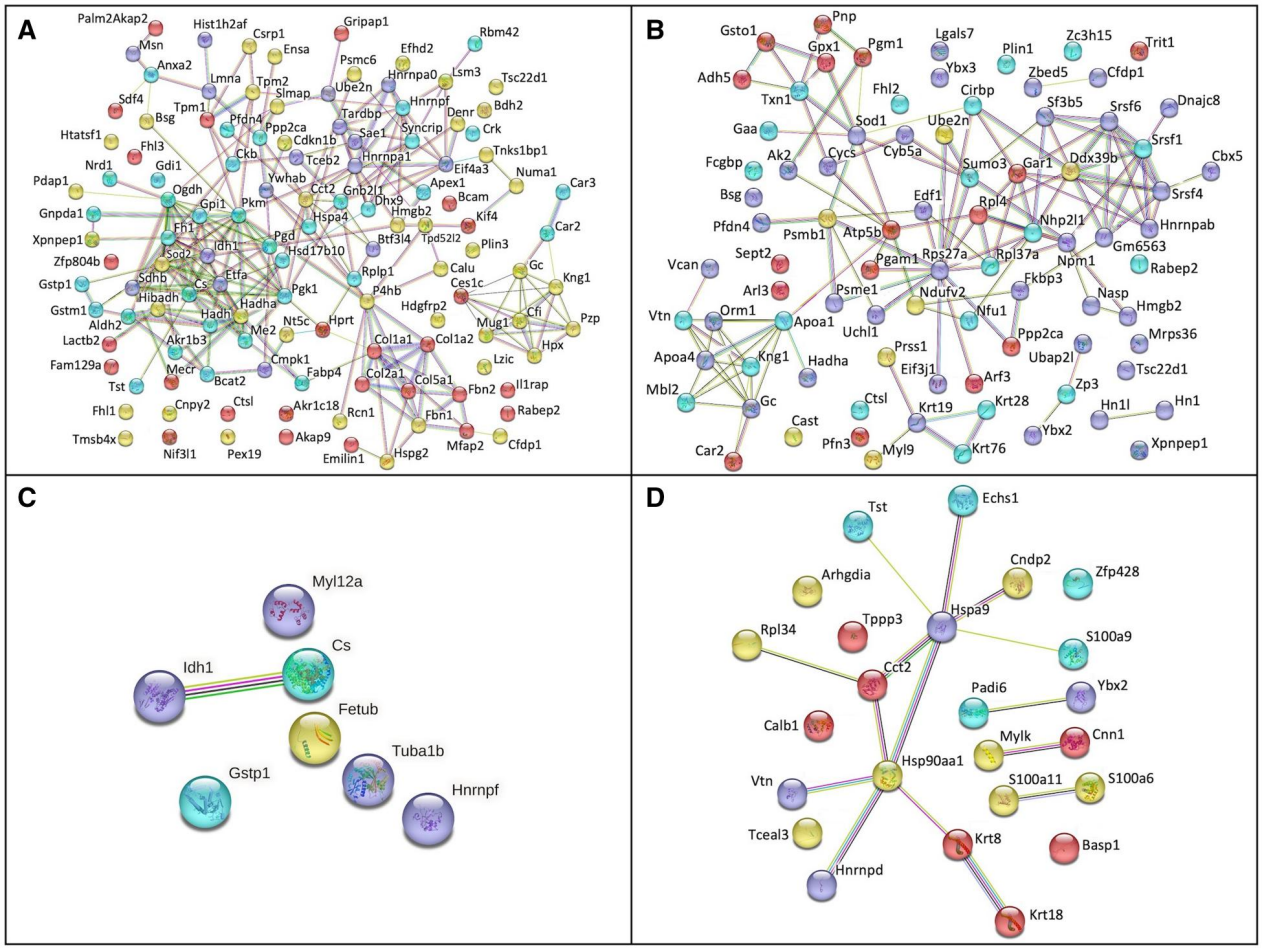
Stringdb interaction plots of proteins altered by DMBA exposure in lean and HFHS-fed mice. Protein (*P *<* *0.05) interaction plot where the color of node dictates the range of log2(FC), where red represents proteins with an FC<−1, yellow is −1 < FC<0, purple is 0 < FC<1, and blue is FC>1. String colors determine the likelihood of interactions, where teal and pink are known interactions, green and blue lines are predicted interactions from gene neighborhood and co-occurrence, yellow, black, and purple lines are predicted from text mining, protein homology, and co-expression. (A) LC vs OC, (B) LC vs LD, (C) OC vs OD, (D) LD vs OD.

Stringdb software was utilized to form a protein interaction plot for altered proteins (*P *<* *0.05) within each treatment comparison (Fig. 5). The resulting plot was compared against the Cytoscape biological processes graphs to look for commonalities in protein clustering. The string plot was also color-coded to observe whether proteins within clusters would be altered in similar or opposing directions. The string plot for basal obesity effects (LC vs OC) contains an upregulated cluster in the top right associated with CRD-mediated mRNA stabilization and positive regulation of cytoplasmic translation. Biological processes associated with cellular metabolism form a tightly interwoven upregulated cluster in the center left of the string plot, whereas proteins involved in the response to reactive oxygen species form an upregulated branch surrounding the bottom of the cellular metabolism cluster. The collagen fibril organization biological process forms a downregulated cluster in the bottom right of the plot. However, proteins involved in negative regulation of intracellular protein transport, such as PPP2CA and GDP Dissociation Inhibitor 1 (GDI1), are unconnected within the plot (Fig. 5A). In lean mice exposed to DMBA (LC vs LD), proteins associated with regulation of cholesterol absorption and hydrogen peroxide metabolic process within the biological processes graph form a common upregulated cluster in the bottom left corner of the string plot. However, proteins involved in negative regulation of calcium ion transport, such as Serine/Threonine-Protein Phosphatase 2A (PPP2CA), Versican (VCAN), and glutathione *S*-transferase omega 1 (GSTO1), appear far apart from each other (Fig. 5B). In obese mice exposed to DMBA (OC vs OD), there is a single upregulated protein interaction between Isocitrate Dehydrogenase 1 (IDH1) and Citrate Synthase within the string plot (Fig. 5C). In DMBA-exposed lean compared with obese mice (LD vs OD), telomerase activity-related proteins form an interactive branch in the string plot. However, proteins associated with the wound-healing biological process do not have any direct interactions within the string plot (Fig. 5D).

## Discussion

The female reproductive system is dynamic and highly conserved such that once the ovarian reserve is depleted, ovarian senescence results (Faddy et al. 1992). Several factors, including obesity and ovotoxicant exposures, alter ovarian function resulting in fertility complications and premature ovarian senescence (Silvestris et al. 2018). Association of obesity with reproductive abnormalities such as miscarriage (Malasevskaia et al. 2021), gestational diabetes mellitus, hypertension (Lewandowska et al. 2020), preeclampsia (Abraham and Romani 2022), reduced assisted reproductive therapy success (MacKenna et al. 2017), prolonged conception period, lower pregnancy, and live birth rates (Dağ and Dilbaz 2015) has been demonstrated. The studies demonstrating the impact of obesity on the ovarian capacity to respond to ovotoxicant exposures have been conducted using a mouse hyperphagia model of obesity in which the diet composition does not differ between the obese and their lean counterparts (Ganesan et al. 2014; Nteeba et al. 2014a). The current study hypothesized that a diet-induced obesity model representative of the Western diet (Hooper et al. 2012) would also affect the capacity for ovarian chemical biotransformation basally and in response to DMBA exposure. Thus, an HFHS diet was utilized and body weight was increased in response to increased caloric intake similar to other studies which have correlated HFHS consumption with increased body weight, BMI, obesity (Rakhra et al. 2020), peripheral tissue steatosis, and insulin resistance (Hintze et al. 2018). A similar body weight increase was achieved in the HFHS-fed mice as previously studied in the hyperphagia-induced obese mouse model (Rishi et al. 2023), to facilitate potential comparison between models. Interestingly, in contrast to findings in the hyperphagic mouse model (Nteeba et al. 2014a; Rishi et al. 2023), there was no effect of HFHS-induced obesity on uterus or ovary weight. Also, there was no impact of HFHS-induced obesity on the duration of estrous cyclicity or on E_2_ or P_4_ in circulation. Contrary to previous observations in a hyperphagic obesity mouse model (Nteeba et al. 2014a; Rishi et al. 2023), primordial follicle number in control-treated HFHS-obese mice was higher than the lean counterparts, suggesting impairments to follicular activation. Antral follicle number was higher in the HFHS-obese control treated mice, which could be attributable to ovulation impairments, although the number of CL did not differ between physiological statuses.

The ovotoxic impact of 14 d of DMBA exposure has been demonstrated (Nteeba et al. 2014a, 2014b), however, a goal of this study was to investigate molecular alterations that precede ovarian cell death thus the duration of exposure was shortened to 7 d, and this duration also ensured that the DMBA exposure spanned the length of a mouse estrous cycle. Except for increased hepatic weight in HFHS-OD-exposed relative to the LD-exposed mice, there were no organ weight effects due to DMBA exposure. Exposure to DMBA also did not affect estrous cyclicity or circulating E_2_ or P_4_, which differed from a hyperphagia-induced obesity model in which DMBA exposure tended to reduce E_2_ in those obese mice (Rishi et al. 2023). There was no evidence of follicle loss at any stage of development due to DMBA exposure, again differing from the hyperphagia-induced obesity model (Rishi et al. 2024), demonstrating that the observed molecular changes were not due to lack of the ovarian structures in which they are produced, although there are potential impairments to ovarian cells that are not immediately evident by histological assessment.

As an untargeted approach, ovarian LC-MS/MS was utilized to evaluate the effects of DMBA exposure in the presence and absence of HFHS-induced obesity. The top 5 biological processes identified from HFHS-induced obese vs lean mice without DMBA exposure were “CRD-mediated mRNA stabilization,” “response to reactive oxygen species,” “monosaccharide biosynthetic process,” “small molecule catabolic process,” and “positive regulation of cytoplasmic translation.” The top 4 biological processes identified from DMBA exposure in the ovaries of lean mice were “regulation of cholesterol absorption,” “negative regulation of mRNA metabolic processes,” “negative regulation of calcium ion transporter,” and “hydrogen peroxide metabolic process” with each protein corresponding to one or more processes depending on the predictive protein function. In the DMBA-exposed females, there were only 2 biological processes affected by HFHS-induced obesity which were “telomerase activity” and “positive regulation of wound healing.”

Independent of the liver, the ovary can biotransform xenobiotics due to the action of metabolizing enzymes (Igawa et al. 2009). DMBA undergoes ovarian biotransformation via CYP1B1 to an intermediate epoxide, which undergoes hydroxylation by EPHX1 to a more potent metabolite DMBA-3,4 diol followed by epoxidation by CYP1A1 or CYP1B1 to the ultimate toxicant DMBA-3,4-diol-1,2-epoxide, that is capable of damaging primordial follicles (Igawa et al. 2009) resulting in oocyte depletion and infertility. In addition to the inverse association of obesity with reproductive health, ovaries from a hyperphagia-induced obesity mouse model have altered capacity to biotransform ovotoxicants such as DMBA metabolites in the Phase II biotransformation reaction (Ganesan et al. 2014; Nteeba et al. 2014a). No basal difference in the abundance of ovarian CYP1A1 was noted between lean and HFHS-induced obese females. Protein abundance of CYP1A1 was increased by DMBA exposure in HFHS-induced obese females, but not in lean females. Lack of induction in lean ovaries is surprising but differential abundance due to an obese phenotype is evident, potentially altering DMBA bioactivation by the ovary and leading to greater ovarian toxicity. Beyond its role in ovarian toxicity, CYP1A1 functions in cholesterol and steroid synthesis (Bayoumy et al. 2018) and obesity upregulates the expression of CYP1A1 in animal models (Hakkak et al. 2008). A study conducted among Egyptian women has highlighted the potential influence of CYP1A1 gene polymorphism (6235T < C) on hormonal profiles and folliculogenesis, potentially contributing to an increased susceptibility to polycystic ovarian syndrome (Bayoumy et al. 2018). Despite the known involvement of EPHX1 in the bioactivation of DMBA (Igawa et al. 2009), neither DMBA nor HFHS-induced obesity impacted ovarian EPHX1 protein levels in contrast to a study in which exposure to DMBA for 14 d increased ovarian EPHX1 protein in ovaries of 20-wk-old lean and hyperphagia-induced obese mice (Nteeba et al. 2014a).

Basally ovarian GSTM1 protein was higher in HFHS-induced obese compared with lean females consistent with previous observations in a different obesity model (Nteeba et al. 2014a) and is of concern because GSTM1 catalyzes the conjugation of GSH to electrophilic compounds (Nebert and Vasiliou 2004) such as DMBA metabolites. GSTM1 is involved in intracellular transport (Listowsky et al. 1988), cell signaling (Hayes and Pulford 1995), and isomerization of steroid hormones (Laborde 2010) and negatively regulates proapoptotic proteins during obesity. GSTM is positively correlated with the prognosis of colon and ovarian cancer and is associated with chemotherapeutic drug resistance (Tecza et al. 2015). Additionally, GSTM forms a complex with proapoptotic apoptosis signal-regulating kinase 1 (ASK1) (Cho et al. 2001) which is reduced when a GSTM substrate exposure occurs to facilitate GSH conjugation in the ovary Cho et al. 2001). Increased basal GSTM could reflect a reduction of the GSTM: ASK1 complex in the HFHS-induced obese females but this needs confirmation. Although some similarities in the findings compared with a previous study were noted herein, the lack of induction of CYP1A1 in lean mice, or EPHX1 or GSTM in either mouse group by DMBA exposure and no observation of basally higher EPHX1, suggests a difference in the obesity experimental paradigm (hyperphagia in KK.CG-A^y/^J mice vs diet induced in wild-type C57Bl6/J mice) impact on ovarian chemical biotransformation. It could also be due to the hyperphagia-induced obese mice being older (20 wk of age) than the mice in the current study or the shortened duration of the DMBA exposure that the mice received.

Reports of reactive oxygen species generation as a mode of PAH chemical-induced toxicity have been reported in extra-ovarian tissues (Kim et al. 2024) as well as in the ovary (Luderer 2014). During obesity, ROS are also elevated (de Mello et al. 2018) and induce ovarian DNA damage (Stepniak and Karbownik-Lewinska 2016). Environmental exposures including cigarette smoke (Siddique et al. 2014) and other ovotoxicants (Wang et al. 2012) induce ovarian ROS generation and oxidative stress reduces fecundity (Diamanti-Kandarakis et al. 2017) and accelerates ovarian aging (Avila et al. 2016). DMBA itself induces ROS (Talat et al. 2022) in in vitro cultured preovulatory ovarian follicles, which was preventable by treating with the antioxidant GSH (Tsai-Turton et al. 2007). In this study, HFHS-induced obesity alone decreased both superoxide dismutase 2 (SOD2) and hemopexin (HPX1). Altered SOD levels are reported to be involved in the response to oxidative stress in many organs including the ovary (Avila et al. 2016). Ovarian HPX is present at differential levels in follicular fluid from old compared with young women (Hashemitabar et al. 2014), potentially indicating a role in ovarian aging. Catalase which is an antioxidant enzyme and responsive to ovarian oxidative stress (Hanukoglu 2006) was induced in HFHS-induced obese mouse ovaries, and altered CAT is associated also with accelerated ovarian aging (Avila et al. 2016). In lean mice exposed to DMBA, glutathione peroxidase 1 was decreased, and both SOD1 and thioredoxin were increased, supporting that oxidative stress is a mode of DMBA-induced ovotoxicity. Far fewer ovarian proteins were altered in the ovaries of HFHS-induced obese mice in response to DMBA exposure, however, isocitrate dehydrogenase, an antioxidant enzyme (Si et al. 2024) was increased by DMBA exposure, supporting that oxidative stress is also ongoing in the HFHS-induced obese ovary due to DMBA exposure.

Interestingly, when compared with the proteomic impacts of hyperphagia-induced obesity, there were fewer proteins that were altered by diet-induced obesity alone, or due to DMBA exposure in lean or obese mice. In the hyperphagia-induced obesity model (Rishi et al. 2023), obesity basally altered 224 proteins, compared with 156 in the diet-induced obesity model. DMBA exposure in lean and obese mice impacted 48 and 120 proteins, respectively, in the hyperphagia-induced obese model, but 100 and 12 in this study. The increase in weight gain was similar in both models and the age of the mice was also similar, thus the mode of obesity induction may be contributory. The proteins that were altered by obesity in both models were GC, RPLP0, EMILIN1, HSPG2, CALU, RABEP2, TSC22D1, SLMAP, FH, SERPINA6, ENSA, NAP1L1, SAE1, HPRT1, ELOB, and DENR. Obesity also consistently altered 5 proteins in DMBA-exposed mice, including HNRNPC, HNRNPD, HSPA9, RPL36A, and KCTD12. DMBA exposure in both studies altered TUBA1B, GSTP, and IDH1 in obese mice.

Finally, a switch between key TFs predicted by in silico analysis was noted in lean and HFHS-induced obese ovaries exposed to DMBA. In lean mice, YY1 was predicted to regulate 46 of the ovarian proteins identified to be altered by DMBA exposure and 84 of the proteins that were altered basally by HFHS-induced obesity. YY1 is a member of the GLI-Kruppel class of zinc finger proteins (Rizkallah and Hurt 2009) and involvement in ovarian folliculogenesis, oocyte-granulosa cell communication, maturation, ovulation, embryogenesis, and cell apoptosis has been documented (Griffith et al. 2011). In addition, there is a negative correlation between YY1 level with survival of ovarian cancer patients (Qian et al. 2020). In HFHS-induced obese mice, JDP2 is predicted to regulate 7 proteins that were altered by DMBA exposure. It is a basic leucine zipper activator protein-1 member (Katz et al. 2001) which functions as a cofactor of the NRF2-MAFK complex, by binding to AHR elements to regulate antioxidant and detoxification enzymes (Tanigawa et al. 2013). Related to female reproductive function, JDP2 may be involved in the regulation of GNRH-mediated FSHβ induction (Jonak et al. 2017). Additionally, JDP2 null female mice had early vaginal opening, reduced number of days to their first litter, increased number of pups per litter and produced their last litter at a younger age than the wild-type counterparts. Mice lacking JDP2 also had higher serum FSH and increased pituitary *Fshb* mRNA. Interestingly, they also had higher estradiol and increased numbers of large follicles with multiple layers of granulosa cells per ovary (Jonak et al. 2017). Thus, JDP2 is a potential candidate for involvement in the response to DMBA exposure in ovaries of HFHS-induced obese mice.

## Conclusion

Taken together, this study illustrates that the ovary responds to DMBA exposure differently depending on physiological status. Basally, diet-induced obesity increased liver weight, increased primordial and preantral follicle number, and changed to ovarian proteome in ways that could impair fertility. Neither lean nor HFHS-induced obese mice exposed to DMBA had a change in follicle number, thus the experimental paradigm facilitated the identification of molecular alterations prior to DMBA-induced follicle loss observed with longer dosing duration. Despite a lack of overt phenotypic alterations due to DMBA exposure, the ovarian proteome of diet-induced obese mouse model was identified and DMBA exposure in both lean and obese mice perturbed the ovarian proteome, with notable changes to chemical biotransformation and oxidative stress proteins with a differential impact of the HFHS-induced obese phenotype. Further, it has been noted that the diet-induced obesity model differs from a hyperphagia-induced obesity model with regards to their basal ovarian phenotypic and proteome impacts and the response to DMBA exposure, emphasizing the importance of model choice for investigating differing physiological outcomes. Whether these changes predicate reduced reproductive potential over the female lifespan remains to be determined. In addition, the systemic alterations that occur with obesity may also impact the ovarian response and require further attention.

## Supplementary Material

kfae150_Supplementary_Data

## Data Availability

Data available upon reasonable request to the corresponding author.
