# Supplementary material for: Diet-induced obesity alters the ovarian chemical biotransformation and oxidative stress response proteins both basally and in response to 7,12-dimethylbenz[a]anthracene exposure
Source: Toxicol Sci. 2025 Feb 6;204(1):9–19. doi: 10.1093/toxsci/kfae150 (PMC11879017; doi:10.1093/toxsci/kfae150)
Supplement: kfae150_Supplementary_Data [file kfae150_supplementary_data.docx]

**Supplemental Table 1. Ovarian proteins altered (*P* < 0.05) basally by obesity (LC vs OC)**

| **UniProtID** | **Protein name** | **Abbreviation** | **log2(FC)** |
| --- | --- | --- | --- |
| Q9DCS3 | Enoyl-[acyl-carrier-protein] reductase, mitochondrial | MECR | -5.1125 |
| A0A650C1X1 | Genome polyprotein | N/A | -2.8966 |
| Q3UIW3 | Lysosome-associated membrane glycoprotein 2 | LAMP2 | -2.7943 |
| Q3TX57 | Fibrillar collagen NC1 domain-containing protein | COL1A2 | -2.4557 |
| Q99KR3 | Endoribonuclease LACTB2 | LACTB2 | -2.3168 |
| Q70FJ1 | A-kinase anchor protein 9 | AKAP9 | -2.2294 |
| F6QGP8 | Zinc finger protein 804B | ZFP804B | -2.1736 |
| Q3U538 | Aldo_ket_red domain-containing protein | AKR1C18 | -2.0194 |
| P28481 | Collagen alpha-1(II) chain | COL2A1 | -1.9862 |
| P11087 | Collagen alpha-1(I) chain | COL1A1 | -1.9687 |
| B1AWB9 | Collagen, type V, alpha 1 | COL5A1 | -1.9383 |
| Q8VD04 | GRIP1-associated protein 1 | GRIPAP1 | -1.9382 |
| G3UY13 | Interleukin-1 receptor accessory protein | IL1RAP | -1.8877 |
| Q99PM0 | Microfibril-associated glycoprotein 1 | MFAP2 | -1.6767 |
| E9PWJ6 | NIF3-like protein 1 | NIF3L1 | -1.6454 |
| A0A125T906 | Light chain kappa | IGK | -1.5824 |
| P23953 | Carboxylesterase 1C | CES1C | -1.5812 |
| Q60950 | MYB-1a | YBX1 | -1.457 |
| Q5FWB6 | 60S acidic ribosomal protein P0 | RPLP0 | -1.4393 |
| Q61112 | 45 kDa calcium-binding protein | SDF4 | -1.3637 |
| Q61555 | Fibrillin-2 | FBN2 | -1.3416 |
| A0A087WPW5 | Kinectin | KTN1 | -1.2778 |
| Q3UWH6 | CATL1 protein | CTSL | -1.2639 |
| Q9R069 | Basal cell adhesion molecule | BCAM | -1.257 |
| P33174 | Chromosome-associated kinesin KIF4 | KIF4 | -1.2539 |
| P09813 | Apolipoprotein A-II | APOA2 | -1.2266 |
| Q91WG2 | Rab GTPase-binding effector protein 2 | RABEP2 | -1.198 |
| Q99K41 | EMILIN-1 | EMILIN1 | -1.1718 |
| Q9R059 | Four and a half LIM domains protein 3 | FHL3 | -1.162 |
| Q6TDG6 | Hypoxanthine guanine phosphoribosyl transferase | HPRT | -1.0856 |
| E9Q450 | Tropomyosin alpha-1 chain | TPM1 | -1.0337 |
| F7AA26 | Paralemmin A kinase anchor protein | PAKAP | -1.0332 |
| Q3UW53 | Protein Niban 1 | NIBAN1 | -1.0297 |
| Q3UKW1 | SERPIN domain-containing protein | SERPINA6 | -1.0244 |
| Q6PB40 | Kcp protein | KCP | -1.0077 |
| P28665 | Murinoglobulin-1 | MUG1 | -0.96508 |
| Q9DBG5 | Perilipin-3 | PLIN3 | -0.95781 |
| B2RTB0 | PDGFA associated protein 1 | PDAP1 | -0.92773 |
| P58871 | 182 kDa tankyrase-1-binding protein | TNKS1BP1 | -0.87839 |
| F6VVN1 | Formin-binding protein 1 | FNBP1 | -0.86253 |
| K3W4Q8 | Basigin | BSG | -0.85621 |
| Q6ZWX2 | Thymosin beta | TMSB4X | -0.83296 |
| Q4FJX9 | Superoxide dismutase | SOD2 | -0.82889 |
| Q8K3C3 | Protein LZIC | LZIC | -0.77504 |
| H7BX64 | Sarcolemmal membrane-associated protein | SLMAP | -0.76071 |
| O88271 | Craniofacial development protein 1 | CFDP1 | -0.74652 |
| A0A0A0MQ79 | Proline-rich coiled-coil 2C | PRRC2C | -0.73106 |
| A0A1B0GRU3 | Zinc finger CCCH domain-containing protein 4 | ZC3H4 | -0.71978 |
| P46414 | Cyclin-dependent kinase inhibitor 1B | CDKN1B | -0.71815 |
| Q542X7 | T-complex protein 1 subunit beta | CCT2 | -0.70125 |
| P62311 | U6 snRNA-associated Sm-like protein LSm3 | LSM3 | -0.69841 |
| P60840 | Alpha-endosulfine | ENSA | -0.69517 |
| Q8BMS1 | Trifunctional enzyme subunit alpha, mitochondrial | HADHA | -0.69421 |
| Q99L13 | 3-hydroxyisobutyrate dehydrogenase, mitochondrial | HIBADH | -0.69267 |
| Q6XLQ8 | Calumenin | CALU | -0.68986 |
| A0A1C7CYU3 | Nucleobindin-1 | NUCB1 | -0.68754 |
| Q61554 | Fibrillin-1 | FBN1 | -0.68138 |
| A0A087WR57 | Death-associated protein 1 | DAP | -0.67693 |
| B1B0C7 | Basement membrane-specific heparan sulfate proteoglycan core protein | HSPG2 | -0.67556 |
| Q8BGC0 | HIV Tat-specific factor 1 homolog | HTATSF1 | -0.67482 |
| A0A0G2JGW9 | Far upstream element-binding protein 1 | FUBP1 | -0.66725 |
| Q61129 | Complement factor I | CFI | -0.6533 |
| A2AEX6 | Four and a half LIM domains protein 1 | FHL1 | -0.63948 |
| A2AUD5 | Tumor protein D54 | TPD52L2 | -0.63833 |
| Q05186 | Reticulocalbin-1 | RCN1 | -0.63163 |
| Q3UMU9 | Hepatoma-derived growth factor-related protein 2 | HDGFL2 | -0.62945 |
| Q61838 | Pregnancy zone protein | PZP | -0.62795 |
| Q8QZS9 | Psmc6 protein | PSMC6 | -0.6242 |
| Q3UR33 | Lamina-associated polypeptide 2 | TMPO | -0.62182 |
| Q6S390 | Plectin 4 | PLEC | -0.61961 |
| A6H663 | BCL2-associated athanogene 3 | BAG3 | -0.6156 |
| Q4FJX4 | Csrp1 protein | CSRP1 | -0.58289 |
| P00493 | Hypoxanthine-guanine phosphoribosyltransferase | HPRT1 | -0.57983 |
| Q3UXU0 | TSC22 domain family protein 1 | TSC22D1 | -0.57911 |
| P21614 | Vitamin D-binding protein | GC | -0.57828 |
| A0A668KLV9 | A-kinase anchor protein 12 | AKAP12 | -0.57021 |
| Q8C845 | EF-hand domain-containing protein D2 | EFHD2 | -0.56909 |
| Q9QXT0 | Protein canopy homolog 2 | CNPY2 | -0.5642 |
| Q6PJ18 | Tpm2 protein | TPM2 | -0.52946 |
| Q9CQJ6 | Density-regulated protein | DENR | -0.52797 |
| Q8JZV9 | Dehydrogenase/reductase SDR family member 6 | BDH2 | -0.52418 |
| P30681 | High mobility group protein B2 | HMGB2 | -0.51269 |
| Q8C7C3 | Tropomyosin alpha-3 chain | TPM3 | -0.48941 |
| Q91X72 | Hemopexin | HPX | -0.48302 |
| E9Q7G0 | Nuclear mitotic apparatus protein 1 | NUMA1 | -0.44197 |
| Q8VCI5 | Peroxisomal biogenesis factor 19 | PEX19 | -0.43597 |
| A2A9X5 | 5'(3')-deoxyribonucleotidase, cytosolic type | NT5C | -0.42131 |
| P09103 | Protein disulfide-isomerase | P4HB | -0.39394 |
| Q3UE92 | Xaa-Pro aminopeptidase 1 | XPNPEP1 | -0.36703 |
| O08677 | Kininogen-1 | KNG1 | -0.29374 |
| P62869 | Elongin-B | ELOB | 0.35253 |
| A0A0R4J0Z1 | Protein disulfide-isomerase A4 | PDIA4 | 0.41297 |
| C6EQH3 | Succinate--CoA ligase [GDP-forming] subunit beta, mitochondrial | SUCLG2 | 0.51732 |
| P48678 | Prelamin-A/C | LMNA | 0.54833 |
| Q9CX86 | Heterogeneous nuclear ribonucleoprotein A0 | HNRNPA0 | 0.63496 |
| A2A7Z4 | Transcription factor BTF3 | BTF3L4 | 0.64584 |
| Q99LC5 | Electron transfer flavoprotein subunit alpha, mitochondrial | ETFA | 0.68836 |
| A2A5N2 | Tyrosine 3-monooxygenase/tryptophan 5-monooxygenase activation protein, beta polypeptide | YWHAB | 0.69069 |
| Q5PPQ7 | Coronin | CORO1C | 0.70614 |
| Q9DBP5 | UMP-CMP kinase | CMPK1 | 0.71027 |
| Q3TEK2 | Heat shock cognate 71 kDa protein | HSPA8 | 0.78479 |
| P26041 | Moesin | MSN | 0.80424 |
| Q8R0B4 | TAR DNA-binding protein 43 | TARDBP | 0.85967 |
| Q3UZG3 | Heterogeneous nuclear ribonucleoprotein A3 | HNRNPA3 | 0.87587 |
| Q3TQW3 | Polypyrimidine tract-binding protein 1 | PTBP1 | 0.88965 |
| A2RTT4 | Ubiquitin-conjugating enzyme E2N | UBE2N | 0.89864 |
| P49312 | Heterogeneous nuclear ribonucleoprotein A1 | HNRNPA1 | 0.91299 |
| Q8CGP5 | Histone H2A type 1-F | HIST1H2AF | 0.91949 |
| A2AFK7 | RNA helicase | EIF4A3 | 0.93246 |
| Q9CQA3 | Succinate dehydrogenase [ubiquinone] iron-sulfur subunit, mitochondrial | SDHB | 0.94656 |
| Q9R1T2 | SUMO-activating enzyme subunit 1 | SAE1 | 0.94726 |
| O88844 | Isocitrate dehydrogenase [NADP] cytoplasmic | IDH1 | 0.95447 |
| Q3UJ44 | Macrophage-capping protein | CAPG | 1.0073 |
| P63330 | Serine/threonine-protein phosphatase 2A catalytic subunit alpha isoform | PPP2CA | 1.076 |
| Q8C290 | Heterogeneous nuclear ribonucleoprotein U | HNRNPU | 1.0892 |
| Q544Z7 | DNA-(apurinic or apyrimidinic site) endonuclease | APEX1 | 1.1105 |
| Q542G9 | Annexin | ANXA2 | 1.1265 |
| Q545S0 | Sulfurtransferase | TST | 1.1303 |
| Q3UWL8 | Prefoldin subunit 4 | PFDN4 | 1.1613 |
| Q9DCD0 | 6-phosphogluconate dehydrogenase, decarboxylating | PGD | 1.167 |
| Q3TE63 | Peptidyl-prolyl cis-trans isomerase | PPIA | 1.1791 |
| Q571M2 | Heat shock 70 kDa protein 4 | HSPA4 | 1.2378 |
| Q8BSH9 | Nucleosome assembly protein 1-like 1 | NAP1L1 | 1.2618 |
| P06745 | Glucose-6-phosphate isomerase | GPI | 1.2708 |
| Q5ND51 | V-crk sarcoma virus CT10 oncogene-like protein | CRK | 1.2721 |
| Q9Z2X1 | Heterogeneous nuclear ribonucleoprotein F | HNRNPF | 1.2772 |
| P68040 | Receptor of activated protein C kinase 1 | RACK1 | 1.32 |
| P50396 | Rab GDP dissociation inhibitor alpha | GDI1 | 1.3247 |
| Q61425 | Hydroxyacyl-coenzyme A dehydrogenase, mitochondrial | HADH | 1.3748 |
| A0A494B997 | N-terminal kinase-like protein | SCYL1 | 1.4139 |
| Q3UZI0 | HNRPR protein | HNRNPR | 1.4206 |
| Q91V81 | RNA-binding protein 42 | RBM42 | 1.4272 |
| Q544B1 | Aldedh domain-containing protein | ALDH2 | 1.4295 |
| P52480 | Pyruvate kinase PKM | PKM | 1.4585 |
| Q3TQP7 | Acetyl-CoA acetyltransferase, mitochondrial | ACAT1 | 1.485 |
| Q8BHG1 | Nardilysin | NRDC | 1.5001 |
| A2AFQ2 | 3-hydroxyacyl-CoA dehydrogenase type-2 | HSD17B10 | 1.5633 |
| Q3TCL2 | Aldo_ket_red domain-containing protein | AKR1B3 | 1.5657 |
| P10649 | Glutathione S-transferase Mu 1 | GSTM1 | 1.5725 |
| P09411 | Phosphoglycerate kinase 1 | PGK1 | 1.5838 |
| Q04447 | Creatine kinase B-type | CKB | 1.6175 |
| P00920 | Carbonic anhydrase 2 | CA2 | 1.6307 |
| Q60597 | 2-oxoglutarate dehydrogenase complex component E1 | OGDH | 1.6564 |
| Q3TCQ3 | Pyruvate carboxylase | PCX | 1.682 |
| Q9CZU6 | Citrate synthase, mitochondrial | CS | 1.7115 |
| O88958 | Glucosamine-6-phosphate isomerase 1 | GNPDA1 | 1.7891 |
| Q99L15 | Acot1 protein | ACOT1 | 1.8526 |
| E9QNN1 | RNA helicase | DHX9 | 1.9622 |
| Q8C6E3 | Catalase | CAT | 1.9655 |
| P97807 | Fumarate hydratase, mitochondrial | FH | 2.0578 |
| Q3ULU3 | Branched-chain-amino-acid aminotransferase | BCAT2 | 2.1108 |
| G3UZ48 | Heterogeneous nuclear ribonucleoprotein Q | SYNCRIP | 2.2246 |
| P16015 | Carbonic anhydrase 3 | CA3 | 2.2434 |
| Q99KE1 | NAD-dependent malic enzyme, mitochondrial | ME2 | 2.2631 |
| Q58E35 | Ribosomal protein, large, P1 | RPLP1 | 2.3594 |
| P19157 | Glutathione S-transferase P 1 | GSTP1 | 2.5268 |
| Q3UK61 | Succinyl-CoA:3-ketoacid-coenzyme A transferase | OXCT1 | 2.7132 |
| Q542H7 | Fatty acid-binding protein, adipocyte | FABP4 | 2.7741 |

**Supplemental Table 2. Ovarian proteins altered (*P* < 0.05) by DMBA exposure in lean mice (LC vs LD)**

| **UniProtID** | **Protein name** | **Abbreviation** | **log2(FC)** |
| --- | --- | --- | --- |
| P00920 | Carbonic anhydrase 2 | CA2 | -2.2919 |
| O09131 | Glutathione S-transferase omega-1 | GSTO1 | -2.2278 |
| A0A0R4J2B2 | BTB/POZ domain-containing protein KCTD12 | KCTD12 | -2.0289 |
| Q3U344 | ADP-ribosylation factor | ARF | -1.5772 |
| P63330 | Serine/threonine-protein phosphatase 2A catalytic subunit alpha isoform | PPP2CA | -1.5292 |
| P42208 | Septin-2 | SEPTIN2 | -1.5085 |
| Q80UN9 | tRNA dimethylallyltransferase | TRIT1 | -1.4495 |
| Q543K9 | Purine nucleoside phosphorylase | PNP | -1.4172 |
| D3YZ09 | H/ACA ribonucleoprotein complex subunit | GAR1 | -1.3389 |
| Q564E8 | 60S ribosomal protein L4 | RPL4 | -1.2886 |
| P56480 | ATP synthase subunit beta, mitochondrial | ATP5F1B | -1.2392 |
| Q6P5I3 | S-(hydroxymethyl)glutathione dehydrogenase | ADH5 | -1.2277 |
| A0A3B2WAL8 | Acetyl-CoA acetyltransferase, cytosolic | ACAT2 | -1.1211 |
| Q7TSV4 | Phosphopentomutase | PGM2 | -1.0976 |
| P11352 | Glutathione Peroxidase 1 | GPX1 | -1.0964 |
| Q543P7 | ADP-ribosylation factor-like protein 3 | ARL3 | -1.0433 |
| Q3U7Z6 | Phosphoglycerate mutase | PGAM1 | -1.0321 |
| Q9DAD6 | Profilin-3 | PFN3 | -1.0092 |
| Q6RI64 | Proteasome subunit beta | PSMB1 | -0.99658 |
| Q9CQ19 | Myosin regulatory light polypeptide 9 | MYL9 | -0.79034 |
| Q9Z1R9 | Protease, serine 1 (trypsin 1) | PRSS1 | -0.7885 |
| Q9D6J6 | NADH dehydrogenase [ubiquinone] flavoprotein 2, mitochondrial | NDUFV2 | -0.75832 |
| A0A0R4J0Z1 | Protein disulfide-isomerase A4 | PDIA4 | -0.60618 |
| Q3UWL8 | Prefoldin subunit 4 | PFDN4 | -0.5961 |
| A2RTT4 | Ubiquitin-conjugating enzyme E2N | UBE2N | -0.50688 |
| Q9Z1N5 | Spliceosome RNA helicase Ddx39b | DDX39B | -0.48726 |
| P51125 | Calpastatin | CAST | -0.2556 |
| Q3TWW8 | Serine/arginine-rich splicing factor 6 | SRSF6 | 0.28196 |
| Q3UE92 | Xaa-Pro aminopeptidase 1 | XPNPEP1 | 0.37764 |
| Q3UK30 | RNA-binding protein FUS | N/A | 0.37981 |
| Q642L7 | Ubiquitin-40S ribosomal protein S27a | RPS27A | 0.41755 |
| P06728 | Apolipoprotein A-IV | APOA4 | 0.44601 |
| Q99MD9 | Nuclear autoantigenic sperm protein | NASP | 0.50685 |
| P30681 | High mobility group protein B2 | HMGB2 | 0.53977 |
| P97825 | Jupiter microtubule associated homolog 1 | JPT1 | 0.56259 |
| P08228 | Superoxide dismutase [Cu-Zn] | SOD1 | 0.56811 |
| Q9R0P9 | Ubiquitin carboxyl-terminal hydrolase isozyme L1 | UCHL1 | 0.57117 |
| Q5SQB7 | Nucleophosmin | NPM1 | 0.57279 |
| Q9JKB3 | Y-box-binding protein 3 | YBX3 | 0.58898 |
| P21614 | Vitamin D-binding protein | GC | 0.64838 |
| Q544Z3 | S1 protein D2 | HNRNPAB | 0.65013 |
| Q60590 | Alpha-1-acid glycoprotein 1 | ORM1 | 0.65177 |
| G5E850 | Cytochrome b5 type A | CYB5A | 0.66196 |
| Q3UXU0 | TSC22 domain family protein 1 | TSC22D1 | 0.66382 |
| A0A494BB95 | Eukaryotic translation initiation factor 4C | EIF1A | 0.67261 |
| Q9WTP6 | Adenylate kinase 2, mitochondrial | AK2 | 0.6838 |
| Q3TM10 | HN1-like protein | JPT2 | 0.69932 |
| A2ALF2 | DnaJ (Hsp40) homolog, subfamily C, member 8 | DNAJC8 | 0.70491 |
| Q8BMS1 | Trifunctional enzyme subunit alpha, mitochondrial | HADHA | 0.71292 |
| Q542V3 | Serine/arginine-rich-splicing factor 4 | SRSF4 | 0.71516 |
| B1AQ78 | Keratin 19 | KRT19 | 0.71669 |
| K3W4Q8 | Basigin | BSG | 0.71977 |
| Q5HZK3 | Proteasome (Prosome, macropain) 28 subunit, alpha | PSME1 | 0.72039 |
| Q9JMG1 | Endothelial differentiation-related factor 1 | EDF1 | 0.72812 |
| O88271 | Craniofacial development protein 1 | CFDP1 | 0.74541 |
| Q56A15 | Cytochrome c | CYCS | 0.75004 |
| Q2YDW1 | Eif3j protein | EIF3J | 0.76452 |
| Q923D4 | Splicing factor 3B subunit 5 | SF3B5 | 0.7788 |
| Q61686 | Chromobox protein homolog 5 | CBX5 | 0.80885 |
| Q9ES94 | Cathepsin X | CTSZ | 0.82008 |
| Q3UBU9 | Peptidylprolyl isomerase | FKBP3 | 0.82511 |
| B2RUF0 | Y box protein 2 | YBX2 | 0.84508 |
| O54974 | Galectin-7 | LGALS7 | 0.87149 |
| Q80X50 | Ubiquitin-associated protein 2-like | UBAP2L | 0.87849 |
| D3Z5B1 | Coiled-coil-helix-coiled-coil-helix domain-containing 2-like | CHCHD2L | 0.91808 |
| Q9CQX8 | Alpha-ketoglutarate dehydrogenase component 4 | MRPS36 | 0.92377 |
| Q3UR33 | Lamina-associated polypeptide 2 | TMPO | 0.92536 |
| F6VVN1 | Formin-binding protein 1 | FNBP1 | 0.96065 |
| E9PYH0 | Versican core protein | VCAN | 0.97171 |
| G3UWX9 | Small ubiquitin-related modifier 3 | SUMO3 | 1.0034 |
| P60824 | Cold-inducible RNA-binding protein | CIRBP | 1.0114 |
| P29788 | Vitronectin | VTN | 1.0265 |
| Q3UEK1 | Mannose-binding protein C | MBL2 | 1.0478 |
| P70699 | Lysosomal alpha-glucosidase | GAA | 1.0492 |
| Q3TIV5 | Zinc finger CCCH domain-containing protein 15 | ZC3H15 | 1.1448 |
| P0DP27 | Calmodulin-2 | CALM2 | 1.1573 |
| O08677 | Kininogen-1 | KNG1 | 1.1766 |
| Q91WG2 | Rab GTPase-binding effector protein 2 | RABEP2 | 1.2017 |
| B2RXM7 | SAP domain-containing protein | SARNP | 1.211 |
| F7D0L1 | Pogo transposable element with ZNF domain | POGZ | 1.2252 |
| Q9D0T1 | NHP2-like protein 1 | SNU13 | 1.2731 |
| Q6PB40 | Kcp protein | KCP | 1.2803 |
| A6BLY7 | Keratin, type I cytoskeletal 28 | KRT28 | 1.3361 |
| H7BX95 | Serine/arginine-rich splicing factor 1 | SRSF1 | 1.3548 |
| Q8BPR2 | Synemin | SYNM | 1.3575 |
| Q3UWH6 | CATL1 protein | CTSL | 1.421 |
| Q3TFP8 | Cytochrome b5 heme-binding domain-containing protein | CYB5B | 1.4322 |
| P10639 | Thioredoxin | TXN | 1.5102 |
| Q3UV17 | Keratin, type II cytoskeletal 2 oral | KRT76 | 1.5129 |
| P10761 | Zona pellucida sperm-binding protein 3 | ZP3 | 1.56 |
| Q9QZ23 | NFU1 iron-sulfur cluster scaffold homolog, mitochondrial | NFU1 | 1.5837 |
| A0A5C1IZY9 | Major core protein 4a precursor | A10L | 1.64 |
| A0A087WPW5 | Kinectin | KTN1 | 1.6872 |
| E9Q0B5 | Fc fragment of IgG-binding protein | FCGBP | 1.6932 |
| Q543D7 | Four and a half LIM domains protein 2 | FHL2 | 1.6966 |
| Q3UTI7 | Peptidyl-prolyl cis-trans isomerase | PIN1 | 1.7338 |
| Q5M9N6 | Rpl37a protein | RPL37A | 1.736 |
| Q00623 | Apolipoprotein A-I | APOA1 | 1.7889 |
| Q8CGN5 | Perilipin-1 | PLIN1 | 1.8794 |
| A0A1W2P7A1 | 40S ribosomal protein S12 | RPS12 | 2.038 |

**Supplemental Table 3. Ovarian proteins altered (*P* < 0.05) by DMBA exposure in obese mice (OC vs OD)**

| **UniProtID** | **Protein name** | **Abbreviation** | **log2(FC)** |
| --- | --- | --- | --- |
| A0A125T906 | Light chain kappa | IGK | -1.497 |
| Q3UJS0 | 60S ribosomal protein L8 | RPL8 | -0.92802 |
| Q6YJU2 | GUGU alpha | FETUB | -0.47204 |
| Q6ZWQ9 | Myosin, light chain 12A, regulatory, non-sarcomeric | MYL12A | 0.19507 |
| O88844 | Isocitrate dehydrogenase [NADP] cytoplasmic | IDH1 | 0.41009 |
| P05213 | Tubulin alpha-1B chain | TUBA1B | 0.46771 |
| Q69ZX3 | MKIAA0866 protein | MYH11 | 0.71132 |
| Q9Z2X1 | Heterogeneous nuclear ribonucleoprotein F | HNRNPF | 0.73973 |
| A0A1S6GWH5 | E1 ubiquitin-activating enzyme | UBA1 | 0.98598 |
| Q9CZU6 | Citrate synthase, mitochondrial | CS | 1.1647 |
| Q8CEH8 | Profilin | PFN1 | 1.2604 |
| P19157 | Glutathione S-transferase P 1 | GSTP1 | 1.3132 |

**Supplemental Table 4. Ovarian proteins altered (*P* < 0.05) by HFHS in DMBA exposed mice (LD vs OD)**

| **UniProtID** | **Protein name** | **Abbreviation** | **log2(FC)** |
| --- | --- | --- | --- |
| P12658 | Calbindin | CALB1 | -4.856 |
| A0A0R4J2B2 | BTB/POZ Domain-Containing Protein KCTD12 | KCTD12 | -3.4586 |
| P05784 | Keratin, Type I cytoskeletal 18 | KRT18 | -2.3995 |
| P11679 | Keratin, Type II cytoskeletal 8 | KRT8 | -2.1605 |
| Q91XV3 | Brain Acid Soluble Protein 1 | BASP1 | -1.6838 |
| Q542X7 | CCT-beta | CCT2 | -1.6413 |
| Q9CRB6 | Tubulin Polymerization-Promoting protein 3 | TPPP3 | -1.3844 |
| B2RSH3 | Calponin | CNN1 | -1.0472 |
| Q545I9 | S100 Calcium Binding Protein A6 | S100A6 | -0.93452 |
| A2AEC2 | Transcription Elongation Factor A like 3 | TCEAL3 | -0.8336 |
| Q80Y52 | Heat Shock Protein 90 Alpha 1 | HSP90AA1 | -0.79559 |
| Q99PT1 | Rho GDP Dissociation Inhibitor Alpha | ARHGDIA | -0.68887 |
| Q9D1A2 | Carnosine Dipeptidase 2 | CNDP2 | -0.68329 |
| Q6PDN3 | Myosin Light Chain Kinase | MYLK | -0.66137 |
| Q9D1R9 | Ribosomal Protein L34 | RPL34 | -0.47904 |
| P50543 | S100 Calcium Binding Protein A11 | S100A11 | -0.40904 |
| Q60668 | Heterogeneous Nuclear Ribonucleoprotein D | HNRNPD | 0.31861 |
| Q3U6P5 | Heterogeneous Nuclear Ribonucleoprotein C | HNRNPC | 0.64577 |
| B2RUF0 | Y-Box Binding Protein 2 | YBX2 | 0.64682 |
| P38647 | Heat Shock Protein A Member 9 | HSPA9 | 0.7061 |
| A0A2I3BPG9 | Ribosomal Protein L36a | RPL36A-PS1 | 0.76229 |
| Q9ES94 | Cathepsin Z | CTSZ | 0.8531 |
| P29788 | Vitronectin | VTN | 0.90208 |
| Q8C1M2 | Zinc Finger Protein 428 | ZNF428 | 1.0998 |
| A0A125T906 | Light Chain Kappa | IGK | 1.1228 |
| Q8K3V4 | Protein-Arginine Deiminase Type 6 | PADI6 | 1.1449 |
| Q8BH95 | Enoyl-CoA Hydrotase | ECHS1 | 1.2112 |
| Q545S0 | Sulfurtransferase | TST | 1.3101 |
| Q3UP42 | S100 Calcium Binding Protein A9 | S100A9 | 2.7154 |

**Supplemental Table 5A**

| **Term** | **Associated Genes Found** |
| --- | --- |
| spliceosomal complex assembly | **[Ddx39b, Srsf1, Srsf6]** |
| positive regulation of DNA binding | **[Calm1, Edf1, Hmgb2, Txn1]** |
| intermediate filament cytoskeleton organization | **[Krt19, Krt76, Sod1, Synm]** |
| positive regulation of organ growth | **[Ddx39b, Pin1, Ybx3]** |
| lung morphogenesis | **[Ctsl, Ctsz, Srsf6]** |
| hydrogen peroxide metabolic process | **[Apoa4, Cycs, Gpx1, Sod1]** |
| sterol biosynthetic process | **[Apoa1, Apoa4, Sod1]** |
| secondary alcohol biosynthetic process | **[Apoa1, Apoa4, Sod1]** |
| cholesterol biosynthetic process | **[Apoa1, Apoa4, Sod1]** |
| vasodilation | **[Gpx1, Kng1, Sod1]** |
| regulation of protein dephosphorylation | **[Calm1, Pin1, Ppp2ca, Vcan]** |
| positive regulation of dephosphorylation | **[Calm1, Pin1, Ppp2ca]** |
| positive regulation of protein dephosphorylation | **[Calm1, Pin1, Ppp2ca]** |
| regulation of phosphoprotein phosphatase activity | **[Calm1, Ppp2ca, Vcan]** |
| negative regulation of calcium ion transmembrane transport | **[Calm1, Gsto1, Ppp2ca]** |
| negative regulation of calcium ion transmembrane transporter activity | **[Calm1, Gsto1, Ppp2ca]** |
| negative regulation of RNA catabolic process | **[Cirbp, Hnrnpab, Srsf1, Ybx2]** |
| negative regulation of mRNA metabolic process | **[Cirbp, Hnrnpab, Npm1, Srsf1, Srsf4, Srsf6, Ybx2]** |
| RNA stabilization | **[Cirbp, Srsf1, Ybx2]** |
| negative regulation of RNA splicing | **[Npm1, Srsf4, Srsf6]** |
| negative regulation of mRNA catabolic process | **[Cirbp, Hnrnpab, Srsf1, Ybx2]** |
| mRNA stabilization | **[Cirbp, Srsf1, Ybx2]** |
| negative regulation of mRNA processing | **[Npm1, Srsf4, Srsf6]** |
| negative regulation of mRNA splicing, via spliceosome | **[Npm1, Srsf4, Srsf6]** |
| lipid digestion | **[Acat2, Apoa1, Apoa4]** |
| hydrogen peroxide metabolic process | **[Apoa4, Cycs, Gpx1, Sod1]** |
| intestinal absorption | **[Acat2, Apoa1, Apoa4]** |
| regulation of digestive system process | **[Acat2, Apoa1, Apoa4]** |
| regulation of intestinal absorption | **[Acat2, Apoa1, Apoa4]** |
| sterol biosynthetic process | **[Apoa1, Apoa4, Sod1]** |
| intestinal lipid absorption | **[Acat2, Apoa1, Apoa4]** |
| secondary alcohol biosynthetic process | **[Apoa1, Apoa4, Sod1]** |
| regulation of sterol transport | **[Acat2, Apoa1, Apoa4]** |
| regulation of intestinal lipid absorption | **[Acat2, Apoa1, Apoa4]** |
| intestinal cholesterol absorption | **[Acat2, Apoa1, Apoa4]** |
| cholesterol biosynthetic process | **[Apoa1, Apoa4, Sod1]** |
| regulation of intestinal cholesterol absorption | **[Acat2, Apoa1, Apoa4]** |
| regulation of cholesterol transport | **[Acat2, Apoa1, Apoa4]** |

**Supplemental Table 5B**

| **Term** | **Associated Genes Found** |
| --- | --- |
| response to electrical stimulus | **[Akap12, Akap9, Sod2]** |
| regulation of substrate adhesion-dependent cell spreading | **[Coro1c, Crk, P4hb]** |
| T cell migration | **[Crk, Msn, Plec]** |
| cell adhesion mediator activity | **[Bcam, Bsg, Emilin1]** |
| positive regulation of cold-induced thermogenesis | **[Fabp4, Fh1, Hadh, Mfap2]** |
| translational elongation | **[Eif4a3, Rack1, Rplp1]** |
| embryo implantation | **[Bsg, Fbn2, Mug1, Pzp]** |
| response to acid chemical | **[Akr1b3, Cdkn1b, Col1a1, Col1a2, Gstp1, Plec]** |
| modulation by host of symbiont process | **[Hspa8, Pcx, Tardbp]** |
| respiratory electron transport chain | **[Etfa, Plec, Sdhb, Sod2]** |
| response to testosterone | **[Akr1c18, Gpi1, Msn]** |
| regulation of humoral immune response | **[Cfi, Hpx, Pzp]** |
| regulation of membrane depolarization | **[Fhl1, Rack1, Slmap]** |
| catechol-containing compound metabolic process | **[Akr1b3, Aldh2, Hprt]** |
| catecholamine metabolic process | **[Akr1b3, Aldh2, Hprt]** |
| kinase inhibitor activity | **[Cdkn1b, Rack1, Ywhab]** |
| protein kinase inhibitor activity | **[Cdkn1b, Rack1, Ywhab]** |
| maintenance of protein location | **[Fbn1, Fbn2, Hnrnpu, Tmsb4x, Ywhab]** |
| embryonic eye morphogenesis | **[Fbn1, Fbn2, Mfap2]** |
| response to cAMP | **[Akap9, Apex1, Bsg, Col1a1, Rplp0]** |
| cellular response to cAMP | **[Akap9, Apex1, Rplp0]** |
| regulation of ATP-dependent activity | **[Dhx9, Hnrnpu, Tpm1, Tpm2]** |
| structural constituent of cytoskeleton | **[Lmna, Plec, Tpm1, Tpm2]** |
| sarcomere organization | **[Csrp1, Plec, Tpm1]** |
| protein folding chaperone | **[Cct2, Hspa4, Hspa8]** |
| chaperone-mediated protein folding | **[Cct2, Hspa8, Pex19]** |
| ATP-dependent protein folding chaperone | **[Cct2, Hspa4, Hspa8]** |
| negative regulation of intracellular transport | **[Bag3, Gdi1, Ppp2ca]** |
| negative regulation of intracellular protein transport | **[Bag3, Gdi1, Ppp2ca]** |
| regulation of protein targeting | **[Bag3, Gdi1, Nucb1]** |
| positive regulation of microtubule polymerization or depolymerization | **[Akap9, Cdkn1b, Numa1]** |
| regulation of microtubule polymerization | **[Akap9, Cdkn1b, Numa1]** |
| positive regulation of microtubule polymerization | **[Akap9, Cdkn1b, Numa1]** |
| regulation of protein dephosphorylation | **[Ensa, Ppia, Ppp2ca, Ptbp1, Ywhab]** |
| positive regulation of dephosphorylation | **[Ppia, Ppp2ca, Ptbp1]** |
| positive regulation of protein dephosphorylation | **[Ppia, Ppp2ca, Ptbp1]** |
| tricarboxylic acid cycle | **[Cs, Fh1, Idh1, Ogdh, Sdhb, Suclg2]** |
| dicarboxylic acid metabolic process | **[Cs, Fh1, Idh1, Me2, Ogdh, Pcx, Sdhb]** |
| pyruvate metabolic process | **[Gpi1, Me2, Ogdh, Pcx, Pgk1, Pkm]** |
| purine-containing compound catabolic process | **[Acat1, Hprt, Nt5c]** |
| ribonucleotide catabolic process | **[Acat1, Hprt, Nt5c]** |
| purine nucleotide catabolic process | **[Acat1, Hprt, Nt5c]** |
| purine ribonucleotide catabolic process | **[Acat1, Hprt, Nt5c]** |
| collagen biosynthetic process | **[Col1a1, Col5a1, Emilin1]** |
| replacement ossification | **[Col1a1, Col2a1, Hspg2]** |
| collagen fibril organization | **[Anxa2, Col1a1, Col1a2, Col2a1, Col5a1, Emilin1]** |
| endochondral bone morphogenesis | **[Col1a1, Col2a1, Hspg2]** |
| endochondral ossification | **[Col1a1, Col2a1, Hspg2]** |
| cartilage development involved in endochondral bone morphogenesis | **[Col1a1, Col2a1, Hspg2]** |
| cellular response to heat | **[Bag3, Dhx9, Hspa8]** |
| chaperone-mediated autophagy | **[Bag3, Hspa8, Lamp2]** |
| striated muscle cell apoptotic process | **[Acot1, Bag3, Hspa8]** |
| negative regulation of muscle cell apoptotic process | **[Acot1, Bag3, Hspa8]** |
| regulation of striated muscle cell apoptotic process | **[Acot1, Bag3, Hspa8]** |
| negative regulation of striated muscle cell apoptotic process | **[Acot1, Bag3, Hspa8]** |
| thioester metabolic process | **[Acat1, Acot1, Cs, Ogdh, Oxct1, Suclg2]** |
| adipose tissue development | **[Acat1, Lmna, Oxct1]** |
| tricarboxylic acid cycle | **[Cs, Fh1, Idh1, Ogdh, Sdhb, Suclg2]** |
| nucleoside bisphosphate metabolic process | **[Acat1, Acot1, Cs, Ogdh, Oxct1, Suclg2]** |
| dicarboxylic acid metabolic process | **[Cs, Fh1, Idh1, Me2, Ogdh, Pcx, Sdhb]** |
| purine nucleoside bisphosphate metabolic process | **[Acat1, Acot1, Cs, Ogdh, Oxct1, Suclg2]** |
| acyl-CoA metabolic process | **[Acat1, Acot1, Cs, Ogdh, Oxct1, Suclg2]** |
| ribonucleoside bisphosphate metabolic process | **[Acat1, Acot1, Cs, Ogdh, Oxct1, Suclg2]** |
| succinyl-CoA metabolic process | **[Ogdh, Oxct1, Suclg2]** |
| alternative mRNA splicing, via spliceosome | **[Dhx9, Eif4a3, Hnrnpa1, Hnrnpu, Ptbp1]** |
| negative regulation of nuclear-transcribed mRNA catabolic process, deadenylation-dependent decay | **[Dhx9, Hnrnpu, Syncrip, Ybx1]** |
| regulation of ATP-dependent activity | **[Dhx9, Hnrnpu, Tpm1, Tpm2]** |
| positive regulation of ATP-dependent activity | **[Dhx9, Hnrnpu, Tpm1]** |
| structural constituent of cytoskeleton | **[Lmna, Plec, Tpm1, Tpm2]** |
| regulation of cytoplasmic translation | **[Dhx9, Hnrnpu, Pkm, Syncrip, Ybx1]** |
| positive regulation of cytoplasmic translation | **[Dhx9, Hnrnpu, Pkm, Syncrip, Ybx1]** |
| nuclear-transcribed mRNA catabolic process, deadenylation-dependent decay | **[Dhx9, Hnrnpu, Syncrip, Ybx1]** |
| CRD-mediated mRNA stabilization | **[Dhx9, Hnrnpu, Syncrip, Ybx1]** |
| regulation of nuclear-transcribed mRNA catabolic process, deadenylation-dependent decay | **[Dhx9, Hnrnpu, Syncrip, Ybx1]** |
| small molecule catabolic process | **[Acat1, Akr1c18, Aldh2, Bcat2, Bdh2, Etfa, Gnpda1, Gpi1, Hadh, Hadha, Hibadh, Hsd17b10, Oxct1, Pgd, Pgk1, Pkm]** |
| branched-chain amino acid metabolic process | **[Acat1, Bcat2, Hibadh, Hsd17b10]** |
| cellular amino acid catabolic process | **[Acat1, Bcat2, Etfa, Hibadh, Hsd17b10]** |
| lipid oxidation | **[Acat1, Bdh2, Etfa, Hadh, Hadha, Hsd17b10]** |
| isoleucine metabolic process | **[Acat1, Bcat2, Hsd17b10]** |
| alpha-amino acid catabolic process | **[Acat1, Bcat2, Hibadh, Hsd17b10]** |
| fatty acid catabolic process | **[Acat1, Bdh2, Etfa, Hadh, Hadha, Hsd17b10]** |
| branched-chain amino acid catabolic process | **[Acat1, Bcat2, Hibadh, Hsd17b10]** |
| fatty acid oxidation | **[Acat1, Bdh2, Etfa, Hadh, Hadha, Hsd17b10]** |
| monocarboxylic acid catabolic process | **[Acat1, Bdh2, Etfa, Hadh, Hadha, Hsd17b10, Pgd]** |
| isoleucine catabolic process | **[Acat1, Bcat2, Hsd17b10]** |
| fatty acid beta-oxidation | **[Acat1, Bdh2, Etfa, Hadh, Hadha, Hsd17b10]** |
| glycolytic process through fructose-6-phosphate | **[Gpi1, Pgk1, Pkm]** |
| canonical glycolysis | **[Gpi1, Pgk1, Pkm]** |
| glycolytic process through glucose-6-phosphate | **[Gpi1, Pgk1, Pkm]** |
| NADH regeneration | **[Gpi1, Pgk1, Pkm]** |
| monosaccharide biosynthetic process | **[Akr1b3, Gnpda1, Gpi1, Pcx, Pgd, Pgk1]** |
| monosaccharide catabolic process | **[Gpi1, Pgk1, Pkm]** |
| hexose biosynthetic process | **[Akr1b3, Gnpda1, Gpi1, Pcx, Pgk1]** |
| hexose catabolic process | **[Gpi1, Pgk1, Pkm]** |
| dicarboxylic acid metabolic process | **[Cs, Fh1, Idh1, Me2, Ogdh, Pcx, Sdhb]** |
| nucleotide phosphorylation | **[Cmpk1, Gpi1, Ogdh, Pgk1, Pkm]** |
| glucose catabolic process | **[Gpi1, Pgk1, Pkm]** |
| pyruvate metabolic process | **[Gpi1, Me2, Ogdh, Pcx, Pgk1, Pkm]** |
| nucleoside diphosphate phosphorylation | **[Cmpk1, Gpi1, Ogdh, Pgk1, Pkm]** |
| glucose catabolic process to pyruvate | **[Gpi1, Pgk1, Pkm]** |
| ATP generation from ADP | **[Gpi1, Ogdh, Pgk1, Pkm]** |
| glycolytic process | **[Gpi1, Ogdh, Pgk1, Pkm]** |
| positive regulation of viral process | **[Bsg, Dhx9, P4hb, Ppia]** |
| response to reactive oxygen species | **[Akr1b3, Apex1, Cat, Col1a1, Crk, Gstp1, Hspa8, Rack1, Sod2, Tpm1]** |
| response to increased oxygen levels | **[Cat, Col1a1, Sod2]** |
| response to hyperoxia | **[Cat, Col1a1, Sod2]** |
| response to hydrogen peroxide | **[Akr1b3, Apex1, Cat, Col1a1, Crk, Hspa8, Rack1, Sod2]** |
| regulation of response to oxidative stress | **[Hspa8, P4hb, Ppia, Rack1, Sod2]** |
| cell death in response to oxidative stress | **[Aldh2, Hspa8, P4hb, Ppia, Rack1, Sod2]** |
| glutathione metabolic process | **[Gstm1, Gstp1, Idh1, Sod2]** |
| smooth muscle cell migration | **[Apex1, Crk, Gstp1, Tmsb4x, Tpm1]** |
| response to ethanol | **[Aldh2, Car3, Cat, Gstp1, Hspa8, Oxct1, Sdf4, Sod2]** |
| response to cadmium ion | **[Cat, Cdkn1b, Gpi1, Hspa8, Sod2]** |
| negative regulation of smooth muscle cell proliferation | **[Cdkn1b, Gstp1, Sod2, Tpm1]** |
| regulation of cellular response to oxidative stress | **[Hspa8, P4hb, Ppia, Rack1, Sod2]** |
| vascular associated smooth muscle cell proliferation | **[Cdkn1b, Gstp1, Sod2, Tpm1]** |
| intrinsic apoptotic signaling pathway in response to oxidative stress | **[Aldh2, P4hb, Ppia, Sod2]** |
| regulation of smooth muscle cell migration | **[Apex1, Crk, Gstp1, Tmsb4x, Tpm1]** |
| cellular response to reactive oxygen species | **[Akr1b3, Apex1, Cat, Hspa8, Rack1, Sod2, Tpm1]** |
| regulation of oxidative stress-induced cell death | **[Hspa8, P4hb, Ppia, Rack1, Sod2]** |
| regulation of vascular associated smooth muscle cell proliferation | **[Cdkn1b, Gstp1, Sod2, Tpm1]** |
| negative regulation of smooth muscle cell migration | **[Apex1, Gstp1, Tpm1]** |
| response to L-ascorbic acid | **[Cat, Gstp1, Sod2]** |
| cellular response to hydrogen peroxide | **[Akr1b3, Apex1, Cat, Hspa8, Rack1]** |
| negative regulation of oxidative stress-induced cell death | **[Hspa8, Ppia, Rack1, Sod2]** |
| negative regulation of vascular associated smooth muscle cell proliferation | **[Cdkn1b, Gstp1, Sod2, Tpm1]** |
| regulation of oxidative stress-induced intrinsic apoptotic signaling pathway | **[P4hb, Ppia, Sod2]** |
| prostanoid metabolic process | **[Akr1b3, Akr1c18, Gstm1, Gstp1]** |
| negative regulation of actin filament polymerization | **[Capg, Tmsb4x, Tpm1]** |
| prostaglandin metabolic process | **[Akr1b3, Akr1c18, Gstm1, Gstp1]** |
| alternative mRNA splicing, via spliceosome | **[Dhx9, Eif4a3, Hnrnpa1, Hnrnpu, Ptbp1]** |
| regulation of alternative mRNA splicing, via spliceosome | **[Eif4a3, Hnrnpa1, Hnrnpu, Ptbp1]** |
| negative regulation of nuclear-transcribed mRNA catabolic process, deadenylation-dependent decay | **[Dhx9, Hnrnpu, Syncrip, Ybx1]** |
| viral translation | **[Denr, Dhx9, Ptbp1]** |
| RNA localization | **[Cct2, Dhx9, Eif4a3, Hnrnpa1, Hnrnpa3, Hnrnpu, Tst, Ybx1]** |
| regulation of ATP-dependent activity | **[Dhx9, Hnrnpu, Tpm1, Tpm2]** |
| positive regulation of ATP-dependent activity | **[Dhx9, Hnrnpu, Tpm1]** |
| regulation of RNA stability | **[Apex1, Dhx9, Hnrnpa0, Hnrnpr, Hnrnpu, Syncrip, Tardbp, Ybx1]** |
| cytoplasmic translation | **[Denr, Dhx9, Hnrnpu, Pkm, Rplp0, Rplp1, Syncrip, Ybx1]** |
| regulation of telomere maintenance | **[Cct2, Hnrnpa1, Hnrnpu, Lmna]** |
| RNA destabilization | **[Dhx9, Hnrnpr, Hnrnpu, Syncrip, Tardbp, Ybx1]** |
| negative regulation of translation | **[Dhx9, Eif4a3, Hnrnpr, Hnrnpu, Rack1, Syncrip, Tardbp, Ybx1]** |
| positive regulation of translation | **[Dhx9, Eif4a3, Hnrnpu, Niban1, Pkm, Syncrip, Ybx1]** |
| regulation of mRNA catabolic process | **[Apex1, Dhx9, Eif4a3, Hnrnpa0, Hnrnpr, Hnrnpu, Syncrip, Tardbp, Ybx1]** |
| negative regulation of RNA catabolic process | **[Dhx9, Hnrnpa0, Hnrnpu, Syncrip, Tardbp, Ybx1]** |
| negative regulation of mRNA metabolic process | **[Dhx9, Hnrnpa0, Hnrnpu, Ptbp1, Rbm42, Syncrip, Tardbp, Ybx1]** |
| positive regulation of mRNA metabolic process | **[Dhx9, Eif4a3, Hnrnpr, Hnrnpu, Hspa8, Syncrip, Tardbp, Ybx1]** |
| regulation of mRNA stability | **[Apex1, Dhx9, Hnrnpa0, Hnrnpr, Hnrnpu, Syncrip, Tardbp, Ybx1]** |
| RNA stabilization | **[Dhx9, Hnrnpa0, Hnrnpu, Syncrip, Tardbp, Ybx1]** |
| regulation of mRNA processing | **[Dhx9, Eif4a3, Hnrnpa1, Hnrnpu, Hspa8, Ptbp1, Rbm42]** |
| positive regulation of mRNA catabolic process | **[Dhx9, Hnrnpr, Hnrnpu, Syncrip, Tardbp, Ybx1]** |
| negative regulation of mRNA catabolic process | **[Dhx9, Hnrnpa0, Hnrnpu, Syncrip, Tardbp, Ybx1]** |
| regulation of telomere maintenance via telomere lengthening | **[Cct2, Hnrnpa1, Hnrnpu]** |
| regulation of cytoplasmic translation | **[Dhx9, Hnrnpu, Pkm, Syncrip, Ybx1]** |
| telomere maintenance via telomerase | **[Cct2, Hnrnpa1, Hnrnpu]** |
| regulation of telomere maintenance via telomerase | **[Cct2, Hnrnpa1, Hnrnpu]** |
| mRNA stabilization | **[Dhx9, Hnrnpa0, Hnrnpu, Syncrip, Tardbp, Ybx1]** |
| mRNA destabilization | **[Dhx9, Hnrnpr, Hnrnpu, Syncrip, Tardbp, Ybx1]** |
| positive regulation of cytoplasmic translation | **[Dhx9, Hnrnpu, Pkm, Syncrip, Ybx1]** |
| nuclear-transcribed mRNA catabolic process, deadenylation-dependent decay | **[Dhx9, Hnrnpu, Syncrip, Ybx1]** |
| regulation of mRNA splicing, via spliceosome | **[Eif4a3, Hnrnpa1, Hnrnpu, Hspa8, Ptbp1, Rbm42]** |
| CRD-mediated mRNA stabilization | **[Dhx9, Hnrnpu, Syncrip, Ybx1]** |
| regulation of nuclear-transcribed mRNA catabolic process, deadenylation-dependent decay | **[Dhx9, Hnrnpu, Syncrip, Ybx1]** |

**Supplemental Table 5C**

| **Term** | **Associated Genes Found** |
| --- | --- |
| positive regulation of wound healing | **[Mylk, S100a9, Vtn]** |
| protein folding chaperone | **[Cct2, Hsp90aa1, Hspa9]** |
| RNA-directed DNA polymerase activity | **[Cct2, Hnrnpd, Hsp90aa1]** |
| regulation of telomerase activity | **[Cct2, Hnrnpd, Hsp90aa1]** |
| telomerase activity | **[Cct2, Hnrnpd, Hsp90aa1]** |

**Supplementary Table 5:** **A breakdown of altered biological processes into subcategories with listed proteins (*P* < 0.05).** Term color represents the pie chart category in Figure 5 for which each subcategory belongs. The color of Associated Genes Found text dictates range of log2(FC) where red represents proteins with a fold change (FC) < -1, yellow is -1 < FC < 0, purple is 0 < FC < 1, and blue is FC > 1. A) LC vs LD, B) LC vs OC, C) LD vs OD.

**Supplemental Table 6. In silico analysis of transcription factors predicted to regulate the differentially abundant proteins in the ovary of an obese relative to a lean mouse (LC vs. OC).**

| **Filtered** | **ClusterCode** | **TF** | **NES** | **#Targets** | **#Motifs/Tracks** |
| --- | --- | --- | --- | --- | --- |
| Yes | M1 | YY1 | 5.220 | 84 | 14 |
| Yes | M2 | ZHX2 | 4.380 | 45 | 7 |
| Yes | M3 | JDP2 | 4.215 | 40 | 2 |
| Yes | M4 | SRF | 4.202 | 35 | 4 |
| Yes | M6 | E2FI | 3.909 | 61 | 2 |
| Yes | M8 | ELK3 | 3.656 | 32 | 2 |
| Yes | M10 | SIX1 | 3.580 | 26 | 3 |
| Yes | M11 | CEBPB | 3.507 | 19 | 6 |
| Yes | M13 | KLF6 | 3.350 | 34 | 3 |
| Yes | M14 | NFYB | 3.342 | 67 | 2 |
| Yes | M15 | MTHFD1 | 3.338 | 17 | 1 |
| Yes | M16 | HSF1 | 3.201 | 11 | 1 |
| Yes | M18 | PITX2 | 3.064 | 44 | 1 |

| Filtered | ClusterCode | TF | NES | #Targets | #Motifs/Tracks |
| --- | --- | --- | --- | --- | --- |
| Yes | M1 | YY1 | 6.531 | 46 | 14 |
| Yes | M2 | NFYB | 4.742 | 34 | 8 |
| Yes | M3 | OVOL1 | 4.636 | 10 | 2 |
| Yes | M4 | E2F6 | 4.390 | 26 | 4 |
| Yes | M5 | BACH1 | 4.322 | 21 | 4 |
| Yes | M6 | HOXB4 | 4.174 | 28 | 4 |
| Yes | M8 | SP1 | 4.025 | 53 | 4 |
| Yes | M9 | HES7 | 4.003 | 18 | 5 |
| Yes | M10 | E2F1 | 3.955 | 23 | 5 |
| Yes | M11 | E2F3 | 3.824 | 37 | 3 |
| Yes | M14 | GMEB2 | 3.601 | 10 | 1 |
| Yes | M15 | FOXP1 | 3.506 | 13 | 3 |
| Yes | M17 | HNF4A | 3.269 | 16 | 2 |
| Yes | M21 | GM13152 | 3.090 | 6 | 1 |
| Yes | M22 | CEBPA | 3.067 | 5 | 1 |
| Yes | M23 | HOMEZ | 3.064 | 12 | 1 |
| Yes | M24 | SOX3 | 3.025 | 6 | 1 |

**Supplemental Table 7. In silico analysis of transcription factors predicted to regulate differentially abundant proteins in lean mice exposed to DMBA (LC vs. LD).**

**Supplemental Table 8. In silico analysis of transcription factors predicted to regulate differentially abundant proteins in obese mice exposed to DMBA (OC vs. OD)**

| **Filtered** | **ClusterCode** | **TF** | **NES** | **#Targets** | **#Motifs/Tracks** |
| --- | --- | --- | --- | --- | --- |
| Yes | M1 | YY1 | 6.639 | 7 | 10 |
| Yes | M3 | E2F1 | 5.808 | 5 | 12 |
| Yes | M4 | SRF | 5.351 | 5 | 21 |
| Yes | M5 | SP1 | 5.075 | 10 | 12 |
| Yes | M6 | CEBPA | 4.724 | 8 | 8 |
| Yes | M7 | TFDP1 | 4.697 | 3 | 4 |
| Yes | M8 | NR2E1 | 4.520 | 5 | 3 |
| Yes | M9 | GRHL2 | 4.333 | 6 | 2 |
| Yes | M11 | ZHX3 | 4.213 | 3 | 1 |
| Yes | M16 | FOXP1 | 3.944 | 3 | 2 |
| Yes | M18 | ELK3 | 3.808 | 7 | 9 |
| Yes | M21 | NR2F6 | 3.583 | 9 | 7 |
| Yes | M24 | HNF1B | 3.522 | 3 | 2 |
| Yes | M25 | MYB | 3.522 | 6 | 3 |
| Yes | M27 | YYI | 3.488 | 6 | 6 |
| Yes | M28 | FOXF2 | 3.477 | 3 | 3 |
| Yes | M29 | RORC | 3.474 | 4 | 5 |
| Yes | M1 | YY1 | 6.639 | 7 | 10 |
| Yes | M3 | E2F1 | 5.808 | 5 | 12 |
| Yes | M4 | SRF | 5.351 | 5 | 21 |
| Yes | M30 | POU5F1 | 3.450 | 4 | 2 |
| Yes | M33 | PITX2 | 3.365 | 3 | 3 |
| Yes | M38 | TRP53 | 3.191 | 3 | 1 |
| Yes | M39 | TFAP4 | 3.130 | 2 | 3 |
